# Which assessments are used to analyze neuromuscular control by electromyography after an anterior cruciate ligament injury to determine readiness to return to sports? A systematic review

**DOI:** 10.1186/s13102-021-00370-5

**Published:** 2021-11-08

**Authors:** Angela Blasimann, Irene Koenig, Isabel Baert, Heiner Baur, Dirk Vissers

**Affiliations:** 1 Department of Health Professions, Division of Physiotherapy, Bern University of Applied Sciences, Murtenstrasse 10, 3008 Bern, Switzerland; 2 Department of Rehabilitation Sciences and Physiotherapy, Faculty of Medicine and Health Sciences, University of Antwerp, Campus Drie Eiken, Universiteitsplein 1, 2610 Wilrijk, Belgium

**Keywords:** Knee, Anterior cruciate ligament injuries, ACL, Electromyography, EMG, Rehabilitation, Patient outcome assessment, Neuromuscular control, Return to sports, RTS

## Abstract

**Background:**

Adequate neuromuscular control of the knee could be one element to prevent secondary injuries after an anterior cruciate ligament (ACL) injury. To assess neuromuscular control in terms of time, amplitude and activity, electromyography (EMG) is used. However, it is unclear which assessments using EMG could be used for a safe return to sports (RTS). Therefore, we aimed to summarize EMG-related assessments for neuromuscular control of the knee in adult patients after an ACL injury to decide upon readiness for RTS.

**Methods:**

This systematic review followed guidelines of Preferred Reporting of Items for Systematic Reviews and Meta-Analyses (PRISMA) and Cochrane recommendations. MEDLINE/PubMed, EMBASE, CINAHL, Cochrane Library, Physiotherapy Evidence Database (PEDro), SPORTDiscus and the Web of Science were searched from inception to March 2019 and updated in November 2020. Studies identifying electromyographic assessments for neuromuscular control during dynamic tasks in adult, physically active patients with an anterior cruciate ligament injury were eligible and qualitatively synthesized. Two independent reviewers used a modified Downs and Black checklist to assess risk of bias of included studies.

**Results:**

From initially 1388 hits, 38 mainly cross-sectional, case-controlled studies were included for qualitative analysis. Most studies provided EMG outcomes of thigh muscles during jumping, running or squatting. Outcomes measures described neuromuscular control of the knee in domains of time, amplitude or activity. Risk of bias was medium to high due to an unclear description of participants and prior interventions, confounding factors and incompletely reported results.

**Conclusions:**

Despite a wide range of EMG outcome measures for neuromuscular control, none was used to decide upon return to sports in these patients. Additional studies are needed to define readiness towards RTS by assessing neuromuscular control in adult ACL patients with EMG. Further research should aim at finding reliable and valid, EMG-related variables to be used as diagnostic tool for neuromuscular control. Moreover, future studies should aim at more homogenous groups including adequately matched healthy subjects, evaluate gender separately and use sport-specific tasks.

*Registration* The protocol for this systematic review was indexed beforehand in the International Prospective Register of Systematic Reviews (PROSPERO) and registered as CRD42019122188.

**Supplementary Information:**

The online version contains supplementary material available at 10.1186/s13102-021-00370-5.

## Background

Anterior cruciate ligament (ACL) injuries happen quite frequently and concern athletes (0.15 injuries per 1000 athletic exposures (AEs)) but also the active part of the general population [1, 2]. Most ACL injuries are due to a non-contact, multiplane mechanism [3] and may lead to instability, secondary meniscal injury or even knee osteoarthritis in the long run [4]. Consequently, this injury means several months or even years of physical impairment with wide consequences for the patients concerning return to work, return to activity or return to sport (RTS). RTS rates between 63 and 97% are reported for patients after ACLR [5, 6]. Most elite athletes return to sports return earlier than non-elite athletes [5], on average within 12 months [6]. However, it remains unclear whether this approach is safe [6], omitting further injury, respectively. Athletes after ACLR returning to high-demanding sports (including jumping, pivoting and hard cutting) show a more than fourfold increase in reinjury rates over two years [7]. More than 5% of athletes with an ACLR sustain a re-rupture of the graft [6, 8] in the ipsilateral knee after RTS. The risk for an ACL tear in the contralateral knee is as double as high (11.8%) even five years or longer after an ACLR [8]. Overall, the recurrence rates even after successful ACLR and subsequent rehabilitation are high (29.5% or 1.82/1000 AEs), with a tear of the ACL graft (9.0%), an ACL injury of the opposite leg (20.5%), muscle injuries on the ipsilateral side or even bilateral consequences [9, 10].

It is known that ipsilateral deficits in clinical knee function and knee laxity persist even years after ACLR [11, 12]. ACL patients show altered kinematics and kinetics [13] and different neuromuscular strategies during walking [14], not only in the injured limb but also in the non-affected side [13, 15]. These changes are referred to neuromuscular adaptations due to altered sensorimotor control [16] and are caused by altered afferent inputs to the central nervous system due to the loss of the mechanoreceptors of the native (original) ACL [17]. Current literature regarding in ACL patients emphasises the importance of understanding consequences of ACL injury regarding neuromuscular control and kinematics [18–20]. To describe neuromuscular control in terms of simultaneously activated agonist/antagonist muscle pairs, generalized knee muscle co-contraction parameters are used [21, 22].

In daily clinical practice, physical performance tests batteries including jumps and tests of muscle function [23] are often used to assess neuromuscular control for RTS. However, there is only limited evidence that passing RTS test batteries—interpreted as having achieved adequate levels of mobility, stability, strength, balance, and neuromuscular control for RTS—reduces the risk for a second ACL injury [24]. Moreover, it remains unclear which measures should be used to bring athletes safely back to RTS with a low risk of re-injury [25]. In conclusion, the currently suggested RTS criteria do not seem to be adequate to assess neuromuscular control of the knee joint to judge upon a safe RTS or even competition. Therefore, meaningful, reliable, valid and accurate diagnostic tools for patients with an ACL injury (either treated surgically or conservatively) are needed and may aid clinical decision-making towards a safe RTS following ACLR. Objective measurements of neuromuscular control should include electromyography (EMG) of involved muscles to judge upon quantity, quality and timing of voluntary activation and reflex activity [13, 20, 26]. However, up to date it is unclear which EMG-related measurements for neuromuscular control are used in patients with an ACL injury to decide upon a safe RTS.

### Objectives

The first objective of this systematic review was therefore to summarize the scientific literature regarding EMG-related assessments for neuromuscular control in adult, physically active patients with an ACL injury (either treated surgically or conservatively) during functional tasks. The second aim was to analyze whether these assessments for neuromuscular control were used to decide upon readiness for RTS in these patients.

## Methods

This systematic review was planned, conducted and analyzed according to the guidelines of Preferred Reporting of Items for Systematic Reviews and Meta-Analyses (PRISMA) [27] and followed the recommendations of Cochrane group [28].

The protocol for this systematic review was indexed beforehand in the International Prospective Register of Systematic Reviews (PROSPERO) and got the registration number CRD42019122188.

### Eligibility criteria

To define the relevant key words for the literature search, the Participants-Intervention-Control-Outcome-Study design (PICOS) scheme was used as follows (Table 1).

**Table 1:** Overview of PICOS criteria for key word definitions

Parameter	Criteria
Participants (P)	Adult people (age of 18–65 years) who sustained an ACL injury, either treated conservatively or surgically (repaired with an autograft)
Intervention (I)	Assessment of neuromuscular control, active knee stability, sensorimotor control, active stability of the lower limb or similar during dynamic activities
Control (C)	Uninjured limb/contralateral side or contralateral lower limb of the ACL-injured participant, or a healthy control group
Outcomes (O)	Any EMG-related outcome describing neuromuscular activity/control in domains of time, amplitude etc.; parameters describing EMG activity of lower limb muscles; related to EMG variables, such as amplitude, timing, mean or peak activity, duration of activity, onset and offset/on–off-pattern respectively, pre-activity, latency, reflex response [14, 20]
Study design (S)	Any laboratory or interventional study, cross-sectional or longitudinal such as randomized controlled trials, clinically controlled trials without randomization, laboratory/experimental controlled trials etc

Studies were considered eligible for this systematic review if they met the following inclusion criteria: Study participants—either females, males or both—had to be 18 years or older, suffer from an ACL injury—either treated conservatively or surgically—with a time since injury/surgery of six months at least, be athletes or physically active people who participate in sports activities on a regular basis (as defined by each study, e.g. Tegner Activity Score (TAS) ≥ 3) to get data to decide upon RTS. Moreover, included studies had to have used active or functional tasks such as walking, stair climbing or jumps, applied assessments for neuromuscular control of lower limb muscles using EMG, be original articles published in peer-reviewed, scientific journals in English, German, French, Italian or Dutch, and available as full texts. Exclusion criteria were model-driven approaches, animals or cadavers, comparisons of surgical techniques, passive or non-functional tasks (such as isokinetic measurements for strength and isometric muscle activity), editorials, conference abstracts, book chapters, theses, systematic reviews and meta-analyses.

### Data sources

The search was effectuated from inception until March 2019 and updated in November 27th, 2020 in the electronic databases MEDLINE/PubMed, EMBASE, CINAHL, Cochrane Library, Physiotherapy Evidence Database (PEDro), SPORTDiscus and in the Web of Science. To ensure new articles matching the search terms, e-mail alerts were established from each of the databases if possible [29]. Furthermore, a hand search was done using the reference lists of included articles to identify additional and potentially eligible articles that had been missed in the electronic database searches. The hits from these two additional sources were also screened for eligibility applying the same criteria as for the articles from the database search.

### Search strategy

In all sources, the advanced search mode was used if available. A search matrix combining relevant keywords (if possible MeSH-terms) with the Boolean operators AND and OR was used and customized for searches in all databases if necessary (see Additional file “Search string for MEDLINE/PubMed” 1): “anterior cruciate ligament/anterior cranial cruciate ligament/ACL”; “anterior cruciate ligament injuries/strains and sprains/rupture/tear/injury/deficiency”; “anterior ligament reconstruction/anterior cruciate ligament/surgery/reconstructive surgical procedures/orthopedic procedure/orthopedic procedure/tendon graft/tendon transfer/conservative treatment/non-surgical/rehabilitation/physical therapy modalities/physiotherapy/kinesiotherapy/exercise/instruction/resistance training/neuromuscular training/postoperative care”; “neuromuscular control/neuromuscular activity/sensorimotor control/muscle activity/active stability”; “electromyography/EMG/electromyogram/amplitude/timing/mean activity/peak activity/duration of activity/onset/offset/on–off-pattern/pre-activity/latency/reflex response”. In the updated search, articles were filtered by date of publication, with the aim of including only those published between March 2019 and November 2020.

### Study selection

All hits obtained by the database searches were downloaded to the Rayyan reference management platform [30] and inserted into EndNote (Clarivate Analytics, Philadelphia, USA). Prior to screening, duplicates were removed. Two authors (AB and IK) independently screened title and abstract of the records, one by using the software EndNote (Clarivate Analytics, Philadelphia, USA) and the other with the help of the free software “rayyan” [30]. After screening, full texts of relevant hits were read by the two authors (IK, AB) to decide upon in- or exclusion. If their decisions did not match, discussion took place until consensus was achieved. If consensus would not have been reached, a third author (IB or HB) would have finally decided upon in- or exclusion of the record in question; however, this was not necessary.

### Data collection process and data extraction

After final decision of all studies, data extraction for each eligible study was performed by the first author (AB) with a predefined Microsoft^®^ Excel (Microsoft Corporation, Redmond WA, USA) spreadsheet as piloted form. The first author (AB) extracted necessary information from each article describing the study design, groups measured and their characteristics, the tasks to be fulfilled by all participants, and all EMG-related assessments or methods used to evaluate neuromuscular control. Furthermore, the chosen assessment for neuromuscular control were judged whether they were used to clear the participants for RTS. The second author (IK) checked the extracted data at random. As all included studies provided enough information to be qualitatively analyzed, it was not necessary to contact corresponding authors for obtaining or confirming data.

### Assessment of risk of bias in included studies

The risk of bias of all the included articles was independently assessed by two raters (AB, IK) by using the Downs and Black checklist [31] in a modified form [29, 32]. The following categories were evaluated: (1) reporting bias: objectives/hypothesis, main outcomes, patients’ characteristics, interventions, principal confounders, main findings, estimates of random variability, actual probability values; (2) external validity bias: study subjects/staff/places/facilities representative; (3) internal validity bias: blinding subjects/assessors, data dredging present, different lengths of follow-up/same time period between intervention and outcome for cases and controls, statistical tests/main outcome measures appropriate; (4) selection bias: patients and controls from same population and over same period of time, randomization, allocation concealed, adjustments for confounding, loss to follow-up; and (5) power analysis (see Additional file “Methodological quality assessment” 2). Each question of the categories was scored with 1 or 2 points if the criterium was fulfilled (answer “yes”), zero points if the answer was “no”, “not fulfilled” and an “X” if the criterium was not applicable, e.g. randomization for a case–control or cross-sectional study, “IC” for intrasubject comparison, respectively.

For this systematic review, studies with a total score of 17 or above out of 25 (more than 2/3 of the maximum total score) were considered as being of high methodological quality, showing a “low” risk of bias respectively [29]. Studies which reached 13 to 16 points (more than 50% of the maximum total score) were rated as being of “medium” quality, and total scores below 13 were rated as being of low methodological quality, “high” risk of bias respectively. As the aim of this systematic review was to summarize the applied measures for neuromuscular control, the methodological quality of the included studies was of secondary interest. Therefore, no study was excluded due to a low total score in the risk of bias assessment.

## Results

### Study selection

**Fig. 1:**
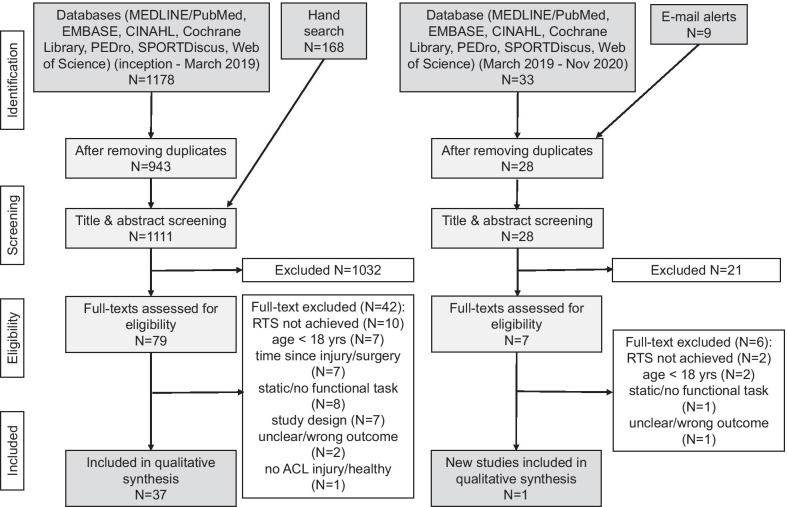
Flowchart of literature search according to guidelines of PRISMA [27]. PEDro = Physiotherapy evidence database; PRISMA = Preferred Reporting of Items for Systematic reviews and Meta-Analyses

Hits from the first and the updated database search including e-mail alerts and hand search were screened for duplicates. After applying in- and exclusion criteria according to PRISMA flowchart [27], a total of 38 articles involving 1236 subjects—809 participants with ACLR or ACL deficiency and 427 healthy controls—could be used for qualitative analysis. Reasons for exclusion were participants younger than 18 years, not able to achieve RTS, time since injury or surgery less than six months, static or non-functional task, study design (e.g. systematic review, study protocol), unclear or inadequate outcome, healthy participants or without ACL injury. Included studies had mainly a cross-sectional, case-controlled study design. Details about every step of the search are illustrated in the following flowchart (Fig. 1).

### Risk of bias assessment

Risk of bias of half (19 studies, 50.0%) of the included studies was medium [13, 33–50], six (15.8%) showed high methodological quality [51–56] and 13 studies (34.2%) were of low quality [57–69] (Table 2). The main reasons for a medium to low methodological quality were due to an unclear description of participants and prior interventions, confounding factors, and incompletely reported results. Table 2 provides details about the risk of bias assessment for each included study.

**Table 2:** Risk of bias assessment with modified Downs and Black checklist [29, 31, 32]

Authors and year	Design	Reporting								External validity			Internal validity												Power	Score	RoB
		1	2	3	4	5	6	7	10	11	12	13	14	15	16	17	18	20	21	22	23	24	25	26	27	Total	Rating
Busch et al. (2019) [13]	CCS	1	1	1	1	2	1	1	1	X	0	1	X	X	1	X	1	1	X	X	X	X	1	X	0	14	Medium
Alkjaer et al. (2003) [33]	CCS	1	1	1	1	2	1	1	1	X	0	1	X	X	1	X	1	1	X	X	X	X	1	X	0	14	Medium
Alkjaer et al. (2002) [34]	CCS	1	1	1	1	2	1	1	1	X	0	1	X	X	1	X	1	1	X	X	X	X	1	1	0	15	Medium
Arnason et al. (2014) [35]	CCS	1	1	1	1	2	1	1	1	X	0	1	X	X	1	X	1	1	1	X	X	X	1	X	0	15	Medium
Bryant et al. (2009) [36]	CCS	1	1	1	1	2	1	1	1	X	0	1	X	X	1	1	1	1	1	X	X	X	1	X	0	16	Medium
Burland et al. (2020) [37]	CSS	1	1	1	1	2	1	1	0	X	X	1	X	X	1	X	1	1	1	X	X	X	1	X	1	15	Medium
Cordeiro et al. (2015) [38]	CCS	1	1	1	1	2	1	1	1	X	0	0	X	X	1	X	1	1	1	X	X	X	X	X	1	14	Medium
Dashti Rostami et al. (2019) [39]	CCS	1	1	1	1	2	1	1	1	X	0	1	X	X	1	1	1	1	X	X	X	X	1	X	0	15	Medium
Jordan et al. (2016) [40]	CCS	1	1	1	1	2	1	1	1	X	0	0	X	X	1	X	1	1	1	X	X	X	X	X	1	14	Medium
Lessi et al. (2017) [41]	CCS	1	1	1	1	2	1	1	0	X	0	1	X	X	1	X	1	1	X	X	X	X	1	X	1	14	Medium
Oliver et al. (2018) [42]	P, CCS	1	1	1	1	1	1	1	1	X	0	1	X	X	1	1	1	1	IC	1	X	X	IC	X	0	14	Medium
Ortiz et al. (2014) [43]	CCS	1	1	1	1	2	1	1	1	X	0	1	X	X	1	X	1	1	1	X	X	X	0	1	0	15	Medium
Patras et al. (2009) [44]	CCS	1	1	1	1	2	1	1	1	X	0	0	X	X	1	0	1	1	IC	X	X	X	IC	X	0	12	Medium
Patras et al. (2010) [45]	CCS	1	1	1	1	2	1	1	1	0	0	0	X	X	1	X	1	1	X	X	X	X	1	X	0	13	Medium
Pincheira et al. (2018) [46]	CCS	1	1	1	1	2	1	1	0	0	0	0	X	X	1	X	1	1	1	X	X	X	X	X	1	13	Medium
Rudolph et al. (2001) [47]	CCS	1	1	1	1	1	1	1	1	1	1	1	X	X	1	X	1	1	1	X	X	X	X	X	0	15	Medium
Rudolph et al. (2000) [48]	CCS	1	1	1	1	1	1	1	1	1	1	1	X	X	1	X	1	1	1	X	X	X	X	X	0	15	Medium
Rudolph and Snyder-Mackler (2004) [49]	CCS	1	1	1	1	1	1	0	1	1	1	0	X	X	1	X	1	1	1	X	X	X	X	X	0	13	Medium
Swanik et al. (2004) [50]	CCS	1	1	1	1	2	1	1	1	X	0	0	X	X	1	X	1	1	0	X	X	X	X	X	1	13	Medium
Briem et al. (2016) [51]	CCS	1	1	1	1	2	1	1	1	1	0	1	X	X	1	X	1	1	1	1	X	X	1	X	1	18	Low
Lessi et al. (2018) [52]	R, CCS	1	1	1	1	2	1	1	1	X	0	1	X	X	1	1	1	1	1	0	X	X	1	X	1	17	Low
Lustosa et al. (2011) [53]	CCS	1	1	1	1	1	1	1	1	1	1	0	X	X	1	1	1	1	1	1	X	X	1	X	0	17	Low
Nyland et al. (2010) [54]	CCS	1	1	1	1	2	1	1	1	1	1	1	X	X	1	1	1	1	0	1	X	X	0	X	0	17	Low
Nyland et al. (2013) [55]	R, CCS	1	1	1	1	2	1	1	1	1	0	1	X	X	1	1	1	1	1	1	X	X	1	X	0	18	Low
Nyland et al. (2014) [56]	CS	1	1	1	1	2	1	1	1	1	0	1	X	X	1	1	1	1	X	1	X	X	1	X	0	17	Low
Boerboom et al. (2001) [57]	CCS	1	1	1	1	2	1	0	0	X	X	1	X	X	1	0	1	0	0	X	X	X	1	X	0	11	High
Bulgheroni et al. (1997) [58]	CCS	1	1	0	1	1	1	0	0	X	X	1	X	X	1	0	1	1	0	0	X	X	X	X	0	9	High
Gokeler et al. (2010) [59]	CCS	1	1	1	1	1	1	1	1	X	0	1	X	X	1	X	1	1	X	X	X	X	X	X	0	12	High
Hansen et al. (2017) [60]	CCS	1	1	1	1	2	1	1	1	X	0	0	X	X	1	X	1	1	X	X	X	X	X	X	0	12	High
Klyne et al. (2012) [61]	CCS	1	1	0	1	1	1	1	1	1	0	1	X	X	1	X	1	1	X	X	X	X	X	X	0	12	High
Knoll et al. (2004) [62]	CCS	1	1	1	1	2	0	0	1	X	X	0	X	X	1	X	1	1	0	X	X	X	X	X	0	10	High
Kuster et al. (1995) [63]	CCS	1	1	1	1	2	1	1	0	X	X	1	X	X	1	X	1	1	0	X	X	X	X	X	0	12	High
Madhavan and Shields (2011) [64]	CCS	1	1	1	1	2	0	0	0	0	0	0	X	X	1	X	1	1	X	X	X	X	X	X	0	9	High
Ortiz et al. (2008) [65]	CCS	1	1	1	1	2	0	0	1	X	0	1	X	X	1	X	1	1	X	X	X	X	0	X	0	11	High
Ortiz et al. (2011) [66]	CCS	1	1	1	1	2	0	0	0	X	0	1	X	X	1	X	1	1	X	X	X	X	0	X	0	10	High
Patras et al. (2012) [67]	CCS	1	1	0	1	2	0	1	0	X	0	0	X	X	0	X	1	1	X	X	X	X	X	X	0	8	High
Swanik et al. (1999) [68]	CCS	1	1	1	1	2	0	1	0	X	0	0	X	X	1	X	1	1	0	X	X	X	X	X	0	10	High
Zebis et al. (2017) [69]	CS	1	1	1	1	2	1	0	0	X	0	1	X	X	1	X	1	1	X	X	X	X	X	X	0	11	High

CCS = Case–control study, CS = case study, IC = intrasubject comparison (injured leg versus healthy leg), P = prospective, R = retrospective (secondary analysis), RoB = risk of bias, X = not applicable or unclear

### Characteristics of included studies

#### Study design

All included studies were case–control studies, except two which where case series [56] or a single-case study [69]. Two reported a retrospective or secondary data analysis [52, 55] or provided a subgroup analysis from a larger trial [45, 47–49, 65–67] (Table 2). Thirty-five studies compared the ACL participants with at least one control group (other ACL treatment, e.g. surgical versus conservative, or healthy controls), the remaining three studies made a comparison between the injured and the non-injured leg of the participants [42, 44] or compared the pre-injury status with follow-up data from pre- and post-surgery [69].

#### Participants

The number of included, adult participants with ACL injury varied from N = 1 [69] to a maximum of N = 70 [62] with a wide range of described physical activity from “normal” [58], “regular” [64], “active in at least one sport” [61], TAS ≥ 3 [50], minimal 2 h/week [33, 34] to athletes at level I sports including jumping, pivoting and hard cutting [42, 57, 59], elite soccer players [35, 38, 67, 69] or elite skiers [50]. Some authors restricted study participation to either males [33, 34, 36, 39, 44–46, 50, 58, 60, 67] or females [50, 51, 64–66, 68, 69], others measured females and males [13, 35, 37, 40, 41, 47–49, 52, 54–57, 59, 61, 62]. Three studies did not provide any data about the gender of their participants [42, 53, 63]. More patient characteristics of included studies can be found in Table 3.

**Table 3:** Participants’ characteristics of included studies

Authors and year	Number of participants (age, sex, group-specific inclusion criteria)	Diagnosis and treatment (only ACL)	Level of activity or sports (RTA, RTS, RTP)	Intervention Group	Control Group 1 (ACL patients)	Control Group 2 (healthy people)	Significant difference between groups?
Busch et al. (2019) [13]	N = 20; N = 10 ACLR (age: 26 ± 10 yrs; height: 175 ± 6 cm; mass: 75 ± 14 kg) and N = 10 healthy matched controls (age: 31 ± 7 yrs; height: 175 ± 7 cm; mass: 68 ± 10 kg)	N = 10 ACLR (13.2 ± 2 months since repair), quadriceps tendon graft by same surgeon, some with additional injuries which needed surgery	TAS min. 4	N = 10 ACLR participants; age 26 ± 10 yrs, height: 175 ± 7 cm, weight: 75 ± 14 kg, 3 females and 7 males, TAS 7 ± 2	n.a.	N = 10 healthy participants without prior injury of the knee, age 31 ± 7 yrs, height 175 ± 8 cm, weight 68 ± 10 kg, 3 females and 7 males, TAS 6 ± 1; matched according to age, height, weight, gender, (sports) activity level and leg dominance	No
Alkjaer et al. (2003) [33]	N = 29; N = 19, all male, complete chronic (post-injury time 6 months or more) ACLD and N = 10 healthy males as controls for EMG	Complete chronic ACLD, min. 6 months of rehab program after injury, ACL injury clinically diagnosed by experienced orthopedic surgeons with Lachman, Anterior Drawer and Pivot-Shift Tests; TLS scores applied to separate ACLD-participants in copers and non-copers	Min. 2 h/wk of physical activity	N = 9; male copers; (mass: 76.7 (14.3) kg, height: 1.81 (0.06) m, age: 28.3 (6.1) yrs); mean TLS scores: 87.1 (5.8) and 6.1 (0.6) respectively; mean time after injury: 39.1 (42.3) (range 6.0–120.0) months	N = 10; male non-copers; mass: 80.4 kg (SD 6.7); height: 1.79 m (SD 0.05), age: 31.7 yrs (SD 5.9); mean TLS scores: 74.0 (SD 7.1) and 3.8 (SD 0.6), respectively; mean time after injury: 55.0 months (SD 42.7) (range 6.0–144.0)	N = 10; male healthy; mass: 77.5 kg (SD 7.9), height: 1.82 m (SD 0.05), age: 31.0 yrs (SD 2.8)	No
Alkjaer et al. (2002) [34]	N = 23 all male; N = 17 males with complete ACLD, N = 6 healthy controls	Complete ACLD, min. 6 months of rehab program after injury; TLS scores applied to separate ACLD-participants in copers and non-copers	Min. 2 h/wk	N = 8; male copers; weight: 76.6 kg (SD 14.8); height: 1.81 m (SD 0.06), age: 26.0 yrs (SD 4.0); mean TLS scores: 85.5 (SD 5.3) and 6.25 (SD 0.5), respectively, mean time after injury: 34.0 months (SD 39.2) (range 6.0–120.0)	N = 9; male non-copers; weight: 80.6 kg (SD 7.1); height: 1.79 m (SD 0.06), age: 31.2 yrs (SD 6.0); mean TLS scores: 74.0 (SD 7.1) and 3.8 (SD 0.6), respectively; mean time after injury: 51.8 months (SD 44.0) (range 6.0–144.0)	N = 6; male healthy; weight: 73.8 kg (SD 7.9), height: 1.81 m (SD 0.05), age: 31.0 yrs (SD 1)	No
Arnason et al. (2014) [35]	N = 36; N = 18, female and male soccer players with ACLR (post-injury time 1–6 yrs) and N = 18 healthy female and male soccer players from the same team (men’s and women’s top league in Iceland), matched for gender, height, body mass and “involved” side designation, as controls	ACLR: successful return to full participation in soccer; no muscle strain injury in knee flexors in past 3 months, no orthopedic condition excluding from soccer	Full participation in soccer (Icelandic top leagues)	N = 18 ACLR participants in total; N = 8 males, N = 10 females; all participants mean mass: 69.2 (11.8) kg, height: 1.73 (0.09) m, age: 23.7 (3.6) yrs; mean BMI: 23.0 (2.4) kg/m^2^; left/right dominance 2/16; involved/uninvolved is the dominant leg 8/10; time since injury 1–6 yrs	n.a.	N = 18 healthy participants; N = 8 males, N = 10 females; all participants mean mass: 68.6 (11.2) kg, height: 1.73 (0.08) m, age: 20.5 (3.7) yrs; mean BMI: 22.7 (2.0) kg/m2	No
Bryant et al. (2009) [36]	N = 59; N = 10 males with ACLD (18–35 yrs); N = 27 matched males with ACLR (14 with patella tendon graft, 13 with combined ST and gracilis graft); N = 22 matched controls	Cincinnati Knee Rating System (0–100 points); ACLD: full ROM, neg. Lachman, neg. Pivot-Shift; confirmed isolated ACL rupture (arthroscopic) min. 1 yr before testing; same orthopedic surgeon for all ACLR	n.m., but hopping required	N = 10 male with ACLD (18–35 yrs)	N = 27 matched males with ACLR (14 with patella tendon graft, 13 with combined ST and gracilis graft)	N = 22 matched (age, activity level, anthropometrics), healthy controls no history of trauma or disease in either knee and no evidence of abnormality on clinical examination	No
Burland et al. (2020) [37]	N = 36; N = 16 females ACLR, N = 10 males ACLR, N = 8 healthy controls (N = 4 females, N = 4 males)	Unilateral ACLR, 21 subjects with BPTB graft, 5 with hamstrings graft; enrolled in physician-directed rehabilitation program, able to do single limb forward hop	n.m. as criteria for in-/exclusion; minimum TAS of 5 preinjury/after surgery	N = 26 with ACLR, age: 20.2 ± 2.7 yrs, mean time since injury: 2.2 ± 2.7 yrs, TAS preinjury: median 8.0 (range 5.0–10.0), after surgery 7.0 (range 5.0–9.0) points, cleared for unrestricted RTS	n.a.	N = 8 healthy controls, age: 23.3 ± 1.8 yrs, TAS: median 9.0 (range 5.0–10.0) points	No, except age
Cordeiro et al. (2015) [38]	N = 17 males; N = 8 with ACLR and N = 9 healthy controls	ACLR: min. 6 months post-surgery on dominant leg, bone-tendon-bone arthroscopy, no problems at end of physiotherapy phase	Soccer, professional level	N = 8 professional male soccer players (age: 24.6 ± 3.5 yrs, height: 1.83 ± 0.06 m, mass: 77.3 ± 7 kg) with ACLR min. 6 months since surgery	n.a.	N = 9 healthy controls; professional male soccer players (age: 24.0 ± 3.5 yrs, height: 1.76 ± 0.05 m, mass: 72.9 ± 3.5 kg), no knee or leg injuries or previous ACL surgeries	No
Dashti Rostami et al. (2019) [39]	N = 36; N = 12 ACLD, N = 12 ACLR; N = 12 healthy controls; all male athletes	For patients: primary unilateral ACL injury	Athletes, regular sports participation, ACLD = copers	N = 12 males, 18 to 36 months post-ACLR	N = 12 males, 18 to 36 months after ACL rupture (= ACLD, copers); grade 2 or 3 rupture including the following definition of copers: athletes with ACLD for at least 18 months, no symptoms of knee instability during regular sports participation	N = 12 healthy males, matched controls; no knee injury, no knee pain	No
Jordan et al. (2016) [40]	N = 22; N = 11 ACLR, N = 11 control; elite skiing athletes from Canada’s national alpine skiing and skier cross team	ACLR: primary ACL injury, at least 12 months post-surgery, actively competing athletes at the Federation International de Ski World Cup level with full medical clearance to compete	Elite ski racers, TAS 10, competing at international level	N = 11 actively competing ACLR skiers (females, n = 5: age: 23.6 ± 1.8 yrs, mass: 61.0 ± 5.3 kg; males, n = 6: age: 26.5 ± 5.8 yrs, mass: 84.4 ± 9.0 kg; 7 subjects with ST autograft, 1 with BPTB autograft, 3 with cadaver allograft	n.a.	N = 11 matched controls with no history of ACL injury (females, n = 5: age: 21.8 ± 3.2 yrs, mass: 63.7 ± 4.6 kg; males, n = 6: age: 23.3 ± 3.3 yr, mass: 84.7 ± 5.1 kg; active competitors at the international level defined as participation in the Federation International de Ski World Cup circuit	n.m.
Lessi et al. (2017) [41]	N = 40; N = 20 with ACLR, N = 20 healthy controls	ACLR: non-contact ACL injury, unilateral reconstruction of the ACL with no prior history of a contralateral ACL injury, no recent history of an ankle, hip, spine, or contralateral knee injury in the past 12 months; rehabilitation completed, cleared to RTS by both their physician and physical therapist	Recreational sports, meaning aerobic or athletic activity at least 3x/wk	N = 20 with ACLR, 13 males, 7 females, at least 12 months post-surgery, 13 with hamstring ipsilateral autografts, 7 with BPTB ipsilateral autograft	n.a.	N = 20 healthy controls, 13 males, 7 females, no history of any dysfunction or previous joint trauma, no prior history of ACL injury or injury of lower extremity in last 12 months; were matched by age, sex, weight, and current sporting activity type	No
Oliver et al. (2018) [42]	N = 25 ACLD, mean age: 22 ± 4.61 yrs, mean mass: 71.18 ± 10.57 kg, mean height: 177.55 ± 9.69 cm; N = 18 males (72%); N = 2 lost to follow-up due to personal issues, all remaining 23 patients concluded the study (pre-surgery, 4 and 6 months post-surgery for questionnaires, at 6 months for jumps)	Complete ACL tear was based on clinical symptoms, on positive Lachman and pivot shift tests, and was confirmed by magnetic resonance imaging; reconstruction 2–3 months after the injury by same surgeon using BTB-technique	More than 200 h of sports activity per year, including jumping, pivoting and twisting actions	Injured knee	Non-injured knee	n.a.	n.a.
Ortiz et al. (2014) [43]	N = 31 females; N = 15 ACLR, N = 16 healthy females	ACLR: same orthopedic surgeon, same rehabilitation protocol, N = 13 were injured while participating in competitive volleyball at the collegiate or professional level; at least 12 months post-surgery, full RTS allowed (without restrictions) to pre-injury level	Sports-specific physical activities as described by the Activity Rating Scale, scores from 12 to 16, consistent with activities such as running, cutting, decelerating, and pivoting more than 2x/wk = high level of participation	N = 15 ACLR with SG graft, age range: 21–35 yrs (height: 167.71 ± 9.0 cm, body mass: 67.68 ± 11.66 kg), time since surgery was between 12 months and 5 yrs, full RTS allowed (pre-injury level); N = 1 drop-out due to inability to perform tasks	n.a.	N = 16 healthy females, participating in volleyball, basketball, and soccer at the collegiate or intramural sports level, age range: 21–35 yrs, height: 160.50 ± 5.17 cm, body mass: 59.35 ± 10.37 kg	No for age and activity, height and weight
Patras et al. (2009) [44]	N = 9 males with ACLR	ACLR: unilateral ACL tear confirmed by MRI and arthroscopy, BPTB graft within 6 months after injury, same rehabilitation protocol, RTS permitted 6 months post-surgery	Athletes, amateur soccer players, at least TAS 7	N = 9 males with ACLR, mean age: 27.7 ± 3.5 yrs, mean weight: 79.5 ± 7.3 kg, mean height: 178 ± 5.9 cm, mean time since surgery: 19.2 ± 5.7 months, median Lysholm score: 95 (range 94–96), TAS: 8 (range 7–9), resumed their sports activities	n.a.	n.a., non-injured side respectively	n.a.
Patras et al. (2010) [45]	N = 28 males; N = 14 ACLR, N = 14 healthy controls	ACLR: unilateral ACL tear confirmed by MRI and arthroscopy, BPTB graft, performed within 6 months after injury, same surgeon, same rehabilitation, RTS permitted after 6 months post-surgery	Amateur soccer players	N = 14 males with ACLR, mean age: 24.8 ± 5.3 yrs; mean height: 177 ± 5.3 cm, mean weight 77.3 ± 7.5 kg, time since surgery: mean 18.5 ± 4.3 months, pre-injury level of sports participation, median Lysholm score 95 (range 94–100) and TAS 8 (range 7–9)	n.a.	N = 14 healthy males, mean age: 21.7 ± 4.4 yrs; mean height: 180 ± 9.0 cm, mean weight 72.2 ± 8.3 kg, never suffered of any kind of orthopedic or neurological condition; left leg = control leg	n.m.
Pincheira et al. (2018) [46]	N = 50 male soccer players; N = 25 with unilateral ACLR, N = 25 uninjured controls	ACLR: unilateral ACLR with ST-gracilis graft, same surgical team, at least 6 months post surgery; non-contact mechanism during soccer match on the dominant limb	Amateur soccer players, playing at least 2x/wk	N = 25 males with ACLR, age: 28.36 ± 7.87 yrs; weight: 77.56 ± 6.35 kg, height: 169 ± 7 cm, time after surgery: 9 ± 3 months, time between ACL injury and surgery: 3.4 ± 1 months; at time of measurements cleared for full RTS	n.a.	N = 25 healthy males, age: 24.16 ± 2.67 yrs; weight 78.16 ± 5.46 kg, height 172 ± 5 cm; without injury or surgery on lower limb	No
Rudolph et al. (2001) [47]	One component of a larger study; N = 31; N = 10 healthy controls, N = 11 ACLD copers, N = 10 ACLD non-copers	ACLD: full range of motion in both knees, no visible or palpable knee effusion, no symptoms of locking, an uninvolved, healthy knee	Athletes, regular activity in level I sports (involving jumping, pivoting, and hard cutting) and level II sports (involving lateral motions) before injury	N = 11 ACLD copers (2 females, 9 males), age range: 22–43 yrs, mean 30.7 yrs, high-level athletes with ACLD for at least 1 year (confirmed by MRI), any knee instability during regular participation in level I and II sports, no more than one episode of giving way, even during sports, since injury	N = 10 non-copers ACLD (4 females, 6 males), age range: 16–43 yrs, mean 28.1 yrs; more than one episode of giving way since injury, instability during ADL, not returned to sports	N = 10 uninjured individuals, matched by age and activity level to the coper subjects (2 females, 8 men), age range: 23–41 yrs, mean 32.2 yrs)	No (age and joint laxity)
Rudolph et al. (2000) [48]	One component of a larger study; N = 31; N = 10 healthy controls, N = 11 ACLD copers, N = 10 ACLD non-copers	ACLD: full range of motion in both knees, no visible or palpable knee effusion, no symptoms of locking, an uninvolved, healthy knee	athletes, regular activity in level I sports (involving jumping, pivoting, and hard cutting) and level II sports (involving lateral motions) before injury	N = 11 ACLD copers (2 females, 9 males), age range: 22–43 yrs, mean 30.7 yrs, high-level athletes with ACLD for at least 1 year (confirmed by MRI, any knee instability during regular participation in level I and II sports, no more than one episode of giving way, even during sports, since injury	N = 10 non-copers ACLD (4 females, 6 males), age range: 16–43 yrs, mean 28.1; more than one episode of giving way since injury, instability during ADL, not returned to sports	N = 10 uninjured individuals, matched by age and activity level to the coper subjects (2 females, 8 men), age range: 23–41 yrs, mean 32.2 yrs)	n.m.
Rudolph and Snyder-Mackler (2004) [49]	One component of a larger study; N = 31; N = 10 healthy controls, N = 11 ACLD copers, N = 10 ACLD non-copers	ACLD: full range of motion in both knees, no visible or palpable knee effusion, no symptoms of locking, an uninvolved, healthy knee	Athletes, regular activity in level I sports (involving jumping, pivoting, and hard cutting) and level II sports (involving lateral motions) before injury	N = 11 ACLD copers (2 females, 9 males), age range: 22–43 yrs, mean 30.7 yrs, high-level athletes with ACLD for at least 1 year (confirmed by MRI, any knee instability during regular participation in level I and II sports, no more than one episode of giving way, even during sports, since injury	N = 10 non-copers ACLD (4 females, 6 males), age range: 16–43 yrs, mean 28.1 yrs; more than one episode of giving way since injury, instability during ADL, not returned to sports	N = 10 uninjured individuals, matched by sex, age and activity level to the coper subjects (2 females, 8 men), age range: 23–41 yrs, mean 32.2 yrs)	No (age and leg length)
Swanik et al. (2004) [50]	N = 29; N = 12 female ACLD, N = 17 female controls	Complete unilateral ACL tear, at least 1 year after injury, mechanical instability (positive Lachman and Pivot-Shift tests), rehabilitation program completed, no ACL surgery	Minimum TAS of 3	N = 12 females with ACLD, age: 25.2 ± 7.3 yrs, mean time since injury 33.6 ± 5.2 months, TAS 5.4 ± 1.83 points	n.a.	N = 17 healthy females, age: 22.7 ± 4.0 yrs, TAS 5.41 ± 1.5 points	n.m.
Briem et al. (2016) [51]	N = 36; N = 18, female players with ACLR (post-injury time 1–6 yrs) and N = 18 healthy female players from the same team (from Icelandic women’s top league in handball, football, basketball), matched for gender, height, body mass and “involved” side designation, as controls	No information about diagnosis or treatment; exclusion criteria: current musculoskeletal injury, lower limb muscle strain within 3 previous months, not being able to do single-limb hops	ACLR: successful return to competition with their teams; healthy: full participation in soccer (Icelandic top leagues)	N = 18 females, ACLR, recruited via advertisement from teams competing in the top leagues in three team sports handball (n = 5), basketball (n = 4), and football (n = 9)]. In 12 instances, the surgical limb was the individual’s dominant one. Characteristics: mean mass: 67.2 (7.8) kg, height: 1.714 (0.05) m, age: 22.7 (3.5) yrs; mean BMI 22.8 (2.4) kg/m2; involved/uninvolved is the dominant leg 12/18; time since injury 1–6 yrs	n.a.	N = 18 healthy females recruited from the same teams, matched for age, height, weight. Characteristics mean mass: 66.3 (7.1) kg, height: 1.708 (0.05) m, age: 21.5 (2.7) yrs; mean BMI 22.7 (2.2) kg/m2	No
Lessi et al. (2018) [52]	N = 14 ACLR (7 males, 7 females) from study of Lessi et al. (2017) [41]	Non-contact ACL injury; unilateral ACLR with autologous ipsilateral graft at least 12 months before recruitment; undergone a rehabilitation program; returned to sports participation; no contralateral ACL injury	Recreational sports	N = 7 males ACLR, age: 23.90 ± 2.80 yrs, height: 1.80 ± 0.1 m, mass: 83.3 ± 7.8 kg, 3 with BPTB graft, 4 with flexor tendons grafts	N = 7 females ACLR, age: 24.7 ± 5.3 yrs, height: 1.63 ± 0.1 m, mass: 65.9 ± 9.0 kg, 2 with BPTB graft, 5 with flexor tendons grafts	n.a.	No, except men were taller than women (P < 0.001) and performed a higher number of sets of the protocol before becoming fatigued their reconstructed limb (P = 0.006)
Lustosa et al. (2011) [53]	N = 25 ACLR; N = 15 with Cincinnati Knee Rating System (CKRS) > 90 points (full RTS), N = 10 with CKRS < 85 points (limited RTS)	At least 2 yrs post-surgery, same rehabilitation program which allowed full RTS activities 7 months post-surgery	Full RTS allowed, not further specified	N = 10 ACLR with CKRS 77.30 ± 6.14 points, age: 33.4 ± 7.53 yrs, time between injury and surgery 52.20 ± 31.33 months, 3 with associated meniscal injuries, 7 without	N = 15 ACLR with CKRS 96.87 ± 2.75 points, age: 34.5 ± 8.85 yrs, time between injury and surgery 67.3 ± 28.5 months, 3 with associated meniscal injuries, 12 without	n.a.	No
Nyland et al. (2010) [54]	N = 70 ACLR; N = 35 males; N = 35 females, 5.3 ± 3 yrs post-surgery	Minimum of 2 yrs since unilateral primary ACL reconstruction with allografts performed by same surgeon, standard rehabilitation program with sufficient adherence	Met or exceeded standard accepted RTS activity goals of a minimum 85% bilateral equivalence with single-leg hop–for–distance testing and 60°/s isokinetic peak knee extensor and flexor torque testing	N = 35 males with ACLR, age n.m., height: 180.3 ± 6.9 cm, weight: 88.9 ± 13.3 kg, time after surgery 5.6 ± 3.2 yrs	N = 35 females with ACLR, age n.m., height: 166.6 ± 7.1 cm, weight: 68.2 ± 18.9 kg, time after surgery 5.1 ± 2.6 yrs	n.a.	n.m.
Nyland et al. (2013) [55]	N = 70 ACLR; 35 male and 35 females, 5.3 ± 3 yrs after surgery; secondary analysis of Nyland et al., 2010 [54]	Minimum of 2 yrs since unilateral primary ACL reconstruction with allografts performed by same surgeon, standard rehabilitation program with sufficient adherence	Met or exceeded standard accepted RTS activity goals of a minimum 85% bilateral equivalence with single-leg hop–for–distance testing and 60°/s isokinetic peak knee extensor and flexor torque testing	N = 24 ACLR well-trained/frequently sporting, 50% males, age at surgery 29.8 ± 11.4 yrs, height: 172.5 ± 8.6 cm, weight: 77.1 ± 18.2 kg, time post-surgery 5.7 ± 2.8 yrs, IKDC 87.3 ± 11.5	N = 26 ACLR only sporting sometimes, 50% males, age at surgery 33.1 ± 13.5 yrs, height: 171.7 ± 9.7 cm, weight: 79.4 ± 23.2 kg, time post-surgery 5.4 ± 3.1 yrs, IKDC 87.3 ± 11.5	No healthy control group, but N = 20 ACLR highly competitive subjects, 50% males, age at surgery 26.5 ± 9.4 yrs, height: 176.5 ± 9.4 cm, weight: 76.8 ± 13.9 kg, time post-surgery 4.6 ± 3.0 yrs, IKDC 91.0 ± 9.4	No
Nyland et al. (2014) [56]	N = 65 ACLR; 32 male and 33 females, 5.2 ± 2.9 yrs after surgery; subject group assignments were made based on how they responded to the following question: “Compared to prior to your knee injury how capable are you now in performing sports activities”, very capable (group 1 see field for healthy controls), capable (group 2), or not capable (group 3)	Minimum of 2 yrs since unilateral primary ACL reconstruction with allografts performed by same surgeon, standard rehabilitation program with sufficient adherence	Met or exceeded standard accepted RTS activity goals of a minimum 85% bilateral equivalence with single-leg hop–for–distance testing and 60°/s isokinetic peak knee extensor and flexor torque testing	N = 23 “capable = group 2”, 52.2% males, age at surgery 29.3 [95% CI: 24.1, 34.4] yrs, height: 172.8 [168.4, 177.3] cm, weight: 76.8 [68.3, 85.2] kg, time post-surgery 5.4 [4.2, 6.6] yrs, IKDC 87.2 [82.1, 92.4]	N = 22 “not capable = group 3”, 45.5% males, age at surgery 33.6 [95% CI: 26.4, 39.1) yrs, height: 172.1 [167.1, 177.1] cm, weight: 79.7 [68.0, 91.3] kg, time post-surgery 5.2 [3.8, 6.5] yrs, IKDC 78.6 [71.7, 85.5]	No healthy control group, but N = 20 “very capable = group 1”, 50% males, age at surgery 26.5 [95% CI: 21.9, 31.8] yrs, height: 176.5 [170.4, 180.1] cm, weight: 76.8 [67.4, 80.3] kg, time post-surgery 4.6 [2.8, 6.2] yrs, IKDC 91.0 [84.1, 94.6]	No
Boerboom et al. (2001) [57]	N = 20; N = 10 ACLD (5 copers, 5 non-copers), N = 10 controls	ACLD: ACL rupture confirmed by physical examination and arthroscopy, conservative treatment	Before injury: all ACLD participants at level I (of the IKDC score), after injury: level I (all copers), level II and III (non-copers)	N = 5 copers (all males) with ACLD, median age: 32 yrs, range 21–46 yrs, median time between primary injury and gait analysis 39 months (13–67), acting at same level of sports and daily activities (level I) as before the injury	N = 5 non-copers (3 males, 2 females) with ACLD, with functional instability, median age: 27 yrs, range 23–35 yrs, median time between injury and gait analysis 22 months (16–87), acting at lower level (4 at level III, 1 at level II)	N = 10 healthy males, without a history of knee injury, median age was 22 yrs (range 18–24 yrs)	No in patient groups (age, time between injury and gait analysis); in comparison with healthy controls: n.m
Bulgheroni et al. (1997) [58]	N = 30 all males; N = 15 with ACLR, N = 10 with ACLD, N = 5 healthy controls	ACLR: BPTB graft	Normal activity	N = 15 males with ACLR, age 25 ± 3 yrs, time after reconstruction: 17 ± 5 months, normal activity	N = 10 males with ACLD, age 27 ± 6 yrs, mean time after injury: 20.4 months after injury (range 8–48 months), knee instability	N = 5 males, healthy controls, age 28 ± 3 yrs, no history of musculoskeletal pathology	n.m.
Gokeler et al. (2010) [59]	N = 20; N = 9 ACLR patients, N = 11 healthy controls	ACLR: 6 months after surgery, isolated ACL lesion, no major meniscal or cartilage lesion, normal limb alignment, no relevant previous surgery at any other joint of the limbs, same rehab program at same institution, unrestricted RTS allowed after 9 months post-surgery	Level I-II athletes	N = 9 ACLR patients (6 males, 3 females), mean age: 28.4 ± 9.7 yrs, 27 ± 1.5 wk postoperatively (BPTB technique, same surgeon)	n.a.	N = 11 healthy subjects (8 males, 3 females), level I-II athletes,	n.m.
Hansen et al. (2017) [60]	N = 37; N = 18 male patients, N = 19 healthy participants	ACLR: discharged from rehabilitation facility	Ready to return to on-fields sports specific activity	N = 18 male ACLR at the end of their rehabilitation and allowed to running, 7 ± 2 months post-surgery; N = 8 with a BPTB graft, age: 27 ± 7.69 yrs, weight: 80.40 ± 9.44 kg, height: 178.49 ± 7.29 cm; N = 10 with a hamstring graft, age: 26 ± 3.84 yrs, weight: 74.16 ± 7.19 kg, height: 176.89 ± 5.6 cm	n.a.	N = 19 injury-free male controls, age: 35.4 ± 7.8 yrs, weight: 77.6 ± 8.4 kg, height: 179.1 ± 5.6 cm	n.m.
Klyne et al. (2012) [61]	N = 26; N = 15 ACLD, N = 11 healthy controls	ACLD: chronic, unilateral ACL rupture demonstrated with a positive pivot shift and confirmed by orthopedic surgeon, plus a history of subjective stability and a right skill preference in the lower limb, without previous ACL surgery	Active in at least one sport	N = 15 ACLD, 10 males and 5 females, age: 28 ± 7 yrs, average time since injury 34 months (± 17 months), sustained injury while playing sport	n.a.	N = 11 healthy controls, 9 males, 2 females (age: 29 ± 8 yrs), active in at least one sport, no other musculoskeletal problems, right skill preferred in their lower limb, matched for age and activity level	n.m.
Knoll et al. (2004) [62]	N = 76; N = 25 ACLR (pre- and postsurgery), N = 51 healthy controls	No previous injury, no meniscal damage, BPTB graft, rehabilitation program	Non-professional athletes pursuing some sports 2-3x/wk	N = 25 with ACLD (before surgery, later ACLR), 18 males, 7 females; first subgroup: 9 male with acute ACLD (mean age: 29.86 ± 6.52 yrs, mean height: 1.77 ± 0.8 m, mean mass: 81.40 kg ± 9.06 kg); second subgroup: 9 males with chronic ACLD (mean age: 39.70 ± 2.1 yrs, mean height: 1.70 ± 0.21 m, mean mass: 88.1 ± 20.2 kg) and 7 females with chronic ACLD (mean age: 30.31 ± 9.48 yrs, mean height: 1.64 ± 0.32 m, mean mass: 62.0 ± 8.4 kg). The chronic ACLD group was examined an average of 28.2 months after injury (ranging from 24 to 52 months), but before surgery	Same population of ACLD, but after surgery ACLR, measured at wk 6, and 4, 8, and 12 months post-surgery	N = 51 healthy controls, 31 males, 20 females, mean age: 31.70 ± 4.1 yrs, mean height: 1.71 ± 0.12 m, mean mass 72.1 ± 25.2 kg, no pathology that would affect gait, unfamiliar with treadmill walking	n.m.
Kuster et al. (1995) [63]	N = 33; N = 21 with ACLD, N = 12 healthy controls	ACLD: arthroscopically confirmed complete ACL ruptures at least 1 year previously	ACLD: TAS range 6–10 (mean 8.2) before injury and range 3–9 (mean 5.3) after injury; controls: TAS range 4–8 (mean 6.1)	N = 19 with 21 ACLD, mean age: 28.2 yrs (range 19–42 yrs), mean height: 174.1 cm (156–187.6 cm), mean weight: 77.9 kg (50–112 kg), mean time since injury 45 months (range of 12–108 months), mean Lysholm score 82 (range 55–100)	n.a.	N = 12 healthy controls, similar in height and weight, mean height: 171.2 cm; weight 70.8 kg, no lower limb injury	Unclear (similar for height and weight)
Madhavan and Shields (2011) [64]	N = 24 females; N = 12 with ACLR, N = 12 healthy controls	Complete reconstruction of the ACL with BPTB or HS autograft, ability to climb stairs without difficulty, full joint ROM, SR-36, KOOS, IKDC	Regular physical activity, TAS	N = 12 females ACLR, age: 22.4 ± 2.4 yrs, mean time from surgery 3.7 ± 1.8 yrs, weight: 144.1 ± 19 kg, height: 164.5 ± 5.28 cm, TAS (current) 7.1 ± 2.4	n.a.	N = 12 healthy females, no previous history of knee pathology, age: 24.1 ± 3.2 yrs, weight: 136.5 ± 20.3 kg, height: 163.8 ± 7.3 cm, TAS (current) 6.9 ± 2.1; matched to age	No
Ortiz et al. (2008) [65]	N = 28 females; N = 13 ACLR, N = 15 non-injured controls	Not controlled for graft/surgery or rehabilitation protocol (only similarities); at least 1-year post surgery, no multiple surgeries on the same knee	Recreational fitness activities such as jogging, running, and weightlifting, none of the participants formed part of any intercollegiate, varsity, or competitive sport team	N = 14 physically active young women with ACLR (age: 25.4 ± 3.1 yrs; height: 167.5 ± 5.9 cm; body mass: 63.2 ± 6.7 kg; mean time after surgery 7.2 ± 4.2 yrs (1–16 yrs after reconstruction); N = 9 with BPTB graft, N = 3 with gracilis-ST-graft, N = 2 with Achilles tendon graft; N = 1 excluded due to inability to perform tasks	n.a.	N = 15 healthy, noninjured young women from physiotherapy school (age: 24.6 ± 2.6 yrs; height: 164.7 ± 6.5 cm; body mass: 58.4 ± 8.9 kg	n.m.
Ortiz et al. (2011) [66]	N = 28 females; N = 13 ACLR, N = 15 non-injured controls (same group as for Ortiz et al., 2008 [65])	Not controlled for graft/surgery or rehabilitation protocol (only similarities); at least 1-year post surgery, no multiple surgeries on the same knee	Recreational fitness activities such as jogging, running, and weightlifting, none of the participants formed part of any intercollegiate, varsity, or competitive sport team	N = 14 physically active young women with ACLR (age: 25.4 ± 3.1 yrs; height: 167.5 ± 5.9 cm; body mass: 63.2 ± 6.7 kg; mean time after surgery 7.2 ± 4.2 yrs (1 − 16 yrs after reconstruction); N = 9 with BPTB graft, N = 3 with gracilis-ST-graft, N = 2 with Achilles tendon graft; N = 1 excluded due to inability to perform tasks	n.a.	N = 15 healthy, non-injured young women from physiotherapy school (age: 24.6 ± 2.6 yrs; height: 164.7 ± 6.5 cm; body mass: 58.4 ± 8.9 kg	n.m.
Patras et al. (2012) [67]	N = 28 males; N = 14 ACLR and N = 14 healthy controls	ACLR: performed sub-acutely within 6 months after the injury from the same surgeon (range 1 to 4 months), unilateral ACL tear confirmed by MRI and arthroscopy; full RTS allowed 6 months post-surgery	Competitive soccer players	N = 14 ACLR with BPTB autograft, age: 24.8 ± 5.3 yrs, weight: 77.3 ± 7.5 kg, height: 177 ± 5.3 cm, mean time since surgery 18.5 ± 4.3 months; TAS 8 (range 7–9), Lysholm score 95 (range 94–100)	n.a.	N = 14 healthy male controls, age: 21.7 ± 4.4 yrs, weight: 72.2 ± 8.3 kg, height: 180 ± 9.0 cm	n.m.
Swanik et al. (1999) [68]	N = 24 females, mean age: 29.4 ± 10.4 yrs; mean height: 168 + 10.7 cm; mean weight: 61.2 ± 6 kg; N = 6 ACLD, N = 12 ACLR, N = 6 controls	Complete unilateral ACL tear, ACLR: BPTB grafts, testing 6–30 months after surgery, rehabilitation program completed, attempt to previous level of activity	Recreational activity at least for healthy controls, TAS of experimental groups 6.8 ± 1.5 points, Lysholm Knee Scoring Scale of experimental groups 92.9 ± 5.4	N = 6 females with ACLD	N = 12 females with ACLR	N = 6 females, healthy controls, recreational activity, no previous history of knee pathology, dominant limb (leg to kick a ball with)	n.m.
Zebis et al. (2017) [69]	N = 1 female, age: 21 yrs	Non-contact ACL injury (video-recorded) in the right knee during match play, ST-gracilis graft, standardized rehabilitation	Elite soccer player	N = 1 female elite soccer player at high level with no previous history of ACL injury	n.a.	Screening of elite soccer players pre-season	n.a.

ACLD = Anterior cruciate ligament deficiency (conservative/non-surgical treatment); ACLR = anterior cruciate ligament reconstruction/repair (surgery); BPTB = bone-patella-tendon-bone technique for ACLR; CI = confidence interval; IKDC = International Knee Documentation Committee; Level I: sports are described as jumping, pivoting and hard cutting sports; Level II sports: also involve lateral motion, but with less jumping or hard cutting than level I; n.a. = not applicable; n.m. = not mentioned; RTA = return to activity (return to participation); RTS = return to sports; RTP = return to performance; SD = standard deviation; ST = semitendinosus muscle; TAS = Tegner Activity Score; TLS = Tegner and Lysholm Score; TSK = Tampa Scale for Kinesiophobia; vs. = versus; wk = week; yrs = years

Details regarding methodological aspects of all included studies are presented in Table 4 below.

**Table 4:** Characteristics of methods of included studies

Authors and year	Tasks: number of repetitions, duration, frequency	Muscles/legs measured	EMG related outcome measure(s), variables	Direct link to RTS?
Busch et al. (2019) [13]	10 × stair descent, warm-up on treadmill with 5 km/h for 10 min to normalize EMG data, (KOOS, Tegner Activity Score, VAS for pain and general well-being)	VM, VL, BF, ST bilaterally	Normalized root mean squares for each muscle, limb and movement phase (preactivation, weight acceptance, push-off) (%subMVC)	No
Alkjaer et al. (2003) [33]	6 trails of walking across 2 force plates at a speed of 4.5 km/h	VL, VM, ST, BF of injured leg of patients and right leg of healthy controls	Mean amplitudes during weight acceptance (%maxEMG); coactivation between VL and BF (method by Rudolph et al. 2001 [45]) (%maxEMG)	(No) → copers and non-copers
Alkjaer et al. (2002) [34]	15 consecutive forward lunges with recordings from hitting a force plate (rest between trials if wanted)	VL, VM, ST, BF of injured leg of patients and right leg of healthy controls	Peak and mean values of EMG amplitudes (microvolts)	(No) → copers and non-copers
Arnason et al. (2014) [35]	3 trials of Nordic hamstring exercise, 3 trials of TRX hamstring curl exercise; order of exercises was randomized, time	MH, LH bilaterally	Peak normalized muscle activation (%MVIC)	(No) → soccer
Bryant et al. (2009) [36]	ACLD and ACLR: involved limb; healthy controls: both limbs; maximal single limb hop for distance on their involved limb from a standing position. 5 trials with 1 min rest in between trials, landing in a fixed position on the takeoff foot	VL, VM, ST, BF	Timing of the onset of muscle activity relative to IC (onset-IC; ms) and timing of the peak of muscle activity relative to IC (ms)	No
Burland et al. (2020) [37]	Single limb forward hop task, distance of their limb length (tip of the greater trochanter to the tip of the lateral malleolus) → unlimited practice trials, 3 successful trials captured consecutively for each limb (trial = successful when participants landed on force platform and balanced on injured limb for a least 1 s); task performed bilaterally, order of limb testing was randomized	VL bilaterally	Peak muscle activity of the VL: EMG signals from heel strike (defined as 10.0 N) to when PKEM was reached were used for statistical analysis. Mean peak muscle activity obtained from this period of interest across the 3 trials was used Dynamic EMG data recorded during task were then normalized to the peak muscle activity recorded across all trials. Muscle activity onset times of VL relative to PKEM (EMG onset = time of PKEM–time of EMG “on”) were established using the Teager–Kaiser Energy Operator (EMG onset = median + 3SD)	No
Cordeiro et al. (2015) [38]	3 instep soccer kicks with dominant leg, (KOOS, TSK)	RF, VL, VM, BF, ST	Muscle activation during knee extension phase (% MVC)	(No) → soccer, instep kick
Dashti Rostami et al. (2019) [39]	Single leg vertical drop landing; 3 proper trials	GM, AL; only the injured limb of ACLR and ACLD individuals and the dominant limb of controls were tested	Preparatory and reactive muscle activity and coactivation from 100 ms prior to initial contact to 250 ms after contact; mean and peak activity (%MVIC); coactivation of GM:AL (method by Rudolph et al. 2001 [45])	No
Jordan et al. (2016) [40]	80 s repeated squat jump test (jump test) on a dual force plate system	VL, VM, BF, ST	Normalized EMG amplitudes at takeoff, at the 25-ms interval prelanding, and at postlanding for the ACLR limb (affected limb), contralateral limb, and limbs of the control subjects (control limb), (Asymmetry index, jump height of body center of mass)	(No) → fatigue, downhill skiing
Lessi et al. (2017) [41]	Single leg landing before and after fatigue (fatigue protocol: 10 squats, 2 vertical jumps, 20 steps)	VL, BF, Gmax	EMG average amplitude of activation, expressed as a %peak EMG during landing	No
Oliver et al. (2018) [42]	Single leg jump from a 25-cm tall box, with hands on hips and without gaining momentum; five times with each leg (injured/non-injured)	VM, VL, RF, ST, BF	Mean values per each patient, leg, and muscle were considered in the analysis; muscle latency time over time of each muscle was defined as the time from touchdown to peak amplitude of EMG activity (RMS) in each muscle. RMS was normalized at the maximum activity of the muscles (%MVC)	No
Ortiz et al. (2014) [43]	60-cm double legged and a 40-cm single legged drop jumps to assess bilateral and unilateral landing strategies, respectively	VM, VL, RF, MH, LH measured in the involved leg of women with ACLR and the dominant leg of the control subjects	Rectified normalized electromyographic activity of the quadriceps and hamstrings (amplitude and latency) in %maximum contraction; quadriceps/hamstrings electromyographic co-contraction ratio (values between 0 and 1); time to maximum neuromuscular activation (time-to-peak muscle activation) in seconds for hamstring and quadriceps muscle groups	No
Patras et al. (2009) [44]	10 min running at moderate intensity (20% below the lactate threshold) and 10 min running at high intensity (40% above the lactate threshold) on separate occasions separated by a time span of 48 h and completed within 10–12 days; moderate intensity = at 20% below the lactate threshold; high intensity = at 40% above the lactate threshold	VL, BF bilaterally	Values from 15 strides averaged to calculate the mean peak amplitude during stance for each recording period	No
Patras et al. (2010) [45]	10 min running at moderate intensity and 10 min running at high intensity on separate occasions separated by a time span of 48 h; moderate intensity = at 80% of the lactate threshold; high intensity = at 40% of the difference betweenVO2max and lactate threshold	VL bilaterally	EMG amplitude during stance, over time respectively in microvolts	No
Pincheira et al. (2018) [46]	2 destabilizing platforms (1 for each limb) generated a controlled perturbation at the ankle of each participant (30° of inversion, 10° plantarflexion simultaneously) in a weight bearing condition; time between the release and the stop (impact) of the mechanism was 200 ± 10 ms	VM, ST	Muscle activation onset times (ms)	No
Rudolph et al. (2001) [47]	5 trials of walking and jogging with 1-3 min rest intervals between trials	LH, VL, SO, medial head of the gastrocnemius muscles of both limbs	Peak EMG activity; onset and termination of muscular activation; duration of muscular activity; co-contraction (integrals calculated)	(No) → copers and non-copers
Rudolph et al. (2000) [48]	Single leg hops	LH, VL, SO, medial head of the gastrocnemius muscles of both limbs	Peak EMG activity over 30 ms from either the dynamic or maximum isometric trials was used to normalize the EMG data (%MVIC); muscle timing variables, muscle intensity: integrating the linear envelope of the EMG curves over a weight acceptance interval (defined as the range from 100 ms prior to initial contact to the point of peak knee flexion. Muscle co-contraction: using normalized EMG data, between the VL and LH, and VL and medial gastrocnemius	(No) → copers and non-copers
Rudolph and Snyder-Mackler (2004) [49]	Step up and over a 26 cm high step; 10 trials, 5 each with the right and left leg ascending a 26 cm step (higher than a typical step, provide a more challenging condition), EMG collected from landing limb	LH, VL, SO, medial head of the gastrocnemius muscles of both limbs	Peak EMG activity (%max); onset and termination of muscular activation; duration of muscular activity; co-contraction	(No) → copers and non-copers
Swanik et al. (2004) [50]	Landing from a hop: The subject stood on a 20-cm step, balanced momentarily on test limb, and hopped to target placed 30 cm horizontally; knee perturbation (special knee pertubation device, 100 N force on the posterior aspect of the tibia → anterior displacement of the tibia)	VL, VM, MH, LH	Muscle activity before and after landing from a hop (area of integrated EMG recordings), hamstring latency after joint perturbation (reflexive muscle activity in the hamstrings assessed by measuring the onset time after anterior translation of the tibia)	No
Briem et al. (2016) [51]	3 consecutive maximal hops (triple jump, single-limb crossover hop for distance), 2 practice trials, 1 single maximal test trial; same procedure for each limb. ACLR participants started with non-surgical limb, each matched control participant with matched limb	MH, LH	Peak activation of the normalized signal (%MVIC)	No
Lessi et al. (2018) [52]	Single leg drop vertical jump landing before and after fatigue protocol (fatigue protocol: 10 squats, 2 vertical jumps, 20 steps)	VL, GM, Gmax	Mean amplitude of activation during landing (% of the peak RMS obtained during the landing task)	No
Lustosa et al. (2011) [53]	Walking at self-selected speed on a 3 m-walkway with 2 stable platforms and 1 electromechanical balance board that could apply a sudden perturbation (20° tilt in the frontal plane (medial/lateral) → varus stress in the slightly flexed knee, leading to external rotation of the femur (= common etiology of ACL injury))	VL, BF	Co-contraction pre- and postperturbation between groups and limbs (co-contraction levels in the 250 ms before perturbation and in the 250 ms after perturbation periods), %MVIC; muscular co-contraction calculated	(No) → stratification of included patients (full RTS or limited RTS)
Nyland et al. (2010) [54]	Single leg CMJ performance	Gmax, VM, MH, GC	Mean EMG signal amplitudes (%MVIC); EMG activation duration during propulsion and landing phase (ms)	No
Nyland et al. (2013) [55]	Single leg CMJ performance	Gmax, VM, MH, GC	EMG amplitude comparison during single leg CMJ propulsion (Difference = involved—uninvolved lower extremity) (%MVIC)	No
Nyland et al. (2014) [56]	Single leg hop test for distance	Gmax, VM, MH, GM	Standardized EMG amplitudes during single leg hop for distance propulsion [%MVIC involved lower extremity − %MVIC uninvolved lower extremity); standardized EMG amplitudes during single leg hop for distance landing [%MVIC involved lower extremity − %MVIC uninvolved lower extremity]	No
Boerboom et al. (2001) [57]	Walking at normal, slower, and faster than normal speed	VM, VL, BF, ST, GC medialis, GC lateralis; of injured leg (patients)	Deviations of the normative EMG profiles (individual averaged EMG pattern during gait)	(No) → copers and non-copers
Bulgheroni et al. (1997) [58]	At least 5 trials of walking at natural cadence (112 ± 5.1 steps/min), 20-m distance used to reach steady state of walking	VL, RF, BF, ST	Amplitude of EMG activity, EMG normalized to the maximum recorded signal amplitude during a single walking cycle	No
Gokeler et al. (2010) [59]	Single leg hop test for distance (arms behind back, maintained balance for at least 1 s after landing, 3 maximal trials for each limb; (IKDC, Rolimeter device for laxity testing)	Gmax, BF, ST, SM, VM, VL, RF, MG, LG, SO	Mean onset times (= preparatory activity before landing) of the EMG signals of each muscle	No
Hansen et al. (2017) [60]	Running on weight-supporting treadmill ("anti-gravity", Alter G, respectively) at 16 km/h with 6 different body weight conditions from 50% (half weight) to 100% (full weight-bearing) in random order	SM, SL, MG, LG, MH, LH	Soleus, gastrocnemius and hamstring cluster formed, SPM used to analyze entire time-dependent EMG signal, comparison of injured vs. non-injured leg and left vs. right leg; EMG signal normalized to its MVC value during 100% body weight running trials for each participant	No
Klyne et al. (2012) [61]	Controlled single leg hop on each limb (arms behind back, landing position hold for at least 1-2 s), length of the horizontal distance hopped was equal to the measured length of the lower leg; 3 successful trials	MG	Onset and offset of MG activation relative to take-off, during flight and landing, muscle activity (RMS), 7 temporal variables (ms, %activity)	No [59]
Knoll et al. (2004) [62]	Walking on treadmill at least 10 min at a constant speed of 2 km/h	VL, VM, BF, AL	Linear envelope EMG curve determined by root mean square method and normalized to average of peak EMG signal values of six gait cycles → EMG patterns during % of gait cycle	(No) → pre-operatively and follow-up (6 weeks, 4, 8, 12 months post-surgery)
Kuster et al. (1995) [63]	At least 5 trials of each task to obtain at least 10 cycles of EMG data for ensemble average processing; level walking and downhill walking on dismountable slope (6 m length, -19° gradient)	RF, BF, GC	Peak muscular activity at heel strike, just before heel strike; values normalized to subject's individual peak levels	No
Madhavan and Shields (2011) [64]	Single leg squat maneuver with random/unexpected perturbations at the start of the flexion phase (triggered compensatory reflex activity)	VM obliquus, RF, VL, LH, MH of exercised limb (reconstruced leg of ACLR subjects, pseudorandomly selected limb of healthy controls to counterbalance ACLR limbs)	Normalized long latency responses (= difference between the mean EMG of perturbation trials and the mean EMG of unperturbed trials, divided by the mean EMG of the unperturbed trials) between 50 and 200 ms after the onset of perturbation of quadriceps and hamstrings; peak velocity (cm/s); latency of peak LLR (= time to peak EMG activity between 50–200 ms following the perturbation); mean muscle EMG activity (%MVIC) in the 200 ms prior to perturbation, 50–200 ms after the perturbation, and 200–400 ms post perturbation	No
Ortiz et al. (2008) [65]	5 trials of a single legged 40-cm drop jump: standing initially on both feet on the 40-cm platform and then standing on the jumping leg, and then to drop when ready to do so, maximal-effort vertical jump on landing single legged on the center of the force plate, use of arms allowed for balance; 2 trials of a 20-cm up-down hop task, participant stood facing a 20-cm step and performed 10 consecutive jumps up to and down when ready. The 10 consecutives up and down hops composed 1 trial	GM, GMax, RF, LH, MH; dominant leg in noninjured women and reconstructed leg in ACLR women	Quadriceps/hamstring cocontraction ratios (values between 0 and 1; closer to 1 = excellent co-contraction, closer to 0 = poor co-contraction) and normalized EMG activity of lower extremity muscles (values between 0 and 1; effect sizes respectively)	No
Ortiz et al. (2011) [66]	Side-to-side hopping task that consisted of hopping single legged 10 times consecutively from side to side across 2 lines marked 30 cm apart on 2 individual force plates. The task was designated as a side hopping when the hop was to the opposite side of the stance leg and as crossover hopping when the hop was toward the side of the stance leg	GM, GMax, RF, LH, MH; dominant leg in noninjured women and reconstructed leg in ACLR women	Quadriceps/hamstring cocontraction ratios (values between 0 and 1; closer to 1 = excellent co-contraction, closer to 0 = poor co-contraction) and normalized EMG activity of lower extremity (values between 0 and 1; effect sizes respectively)	No
Patras et al. (2012) [67]	2 10-min treadmill runs on 2 occasions in the lab, 1 at a moderate (80%VO2max) and 1 at a high intensity (85–88% VO2max), EMG recordings at the 3rd, 5th, 7th, and 10th minute of the runs	VL, BF bilaterally: left leg of controls selected for analysis	Peak EMG amplitude during the stance phase	No
Swanik et al. (1999) [68]	4 functional activities: downhill walking (15°, 0.92 m/s), level running (2.08 m/s), and hopping (self-paced) and landing from a jump (20.3 cm)	VL, VM, MH, LH	Integrated EMG (microvolts x ms) normalized to mean amplitude of 3–6 consecutive test repetitions → mean area and peak integrated EMG of a 250 ms-period after ground contact = reactive muscle activity; testing order and leg assessed by random	No
Zebis et al. (2017) [69]	Standardized side cutting maneuver, CJM with the hands placed at the hip (akimbo), and maximal jump height was calculated	VL, BF, ST	EMG preactivity	(No) → single case, risk profile retrospective, pre-/post-surgery and post-intervention

AL = adductor longus muscle; BF = biceps femoris muscle; CMJ = countermovement jump(ing); EMG = electromyography; GC = gastrocnemius muscles; GM = gluteus medius muscle; GMax = gluteus maximus muscle; GRF = ground reaction force; Hz = Hertz; LG = gastrocnemius lateral head; LH = lateral hamstring muscle; MG = gastrocnemius medial head; MH = medial hamstring muscle; ms = milliseconds; PKEM = peak knee extension moment; RF = rectus femoris muscle; SL = soleus lateralis muscle; SM = soleus medialis muscle; SO = soleus muscle; SPM = Statistical Parametric Mapping; ST = semitendinosus muscle; VL = vastus lateralis muscle; VM = vastus medialis muscle; vs. = versus; WA = weight acceptance

#### Interventions

The number of muscles assessed ranged from one [37, 45, 61] to ten [59]. Mainly muscle activity of four muscles of the thigh, vastus lateralis, vastus medialis, biceps femoris and semitendinosus, had been assessed. However, there were also studies measuring the adductor longus [39, 62], gluteus medius [39, 65, 66], gluteus maximus [41, 52, 54–56, 59, 65, 66], and calf muscles such as soleus, medial and lateral gastrocnemius [47–49, 54–56, 59, 60, 63].

The tasks used were very diverse: there were activities of daily life such as walking on even ground and downhill [33, 47, 53, 57, 58, 62, 63, 68], and stair climbing [13, 49]. Other activities went more towards sports such as running [44, 45, 60, 67, 68] and jumping [36, 37, 39–42, 48, 50–52, 54–56, 59, 61, 65, 66, 68] where mainly the single-leg hop for distance, drop jumps and countermovement jumps were used. Some authors chose typical rehabilitation exercises such as forward lunges [34], Nordic hamstrings or hamstrings curls [35] and squats [64]. At the other end of the scale, more complex, highly demanding, sport-specific tasks such as an instep soccer kick [38] or a sidecutting maneuver [69] were reported. Only few research groups used perturbation platforms to simulate injury mechanisms during walking [53] or squatting [46, 64], or applied devices to stress the ACL in the posterior-anterior direction [50]. In addition, two studies even investigated the influence of fatigue on neuromuscular control [41, 52].

#### Outcomes

All included studies used surface EMG as method to assess neuromuscular control and provided EMG-related variables such as peak and mean amplitudes, timing and peak of muscle activity, preparatory and reactive muscle activity, on- and offset of muscular activation, co-activation/co-contraction ratios, or asymmetry index. The outcome variables were expressed as percentage of maximum voluntary (isometric) contraction (%MVIC or %MVC) or reported in microvolts or milliseconds according to the variable chosen in amplitude or time domain.

#### Decision for Return to Sports (RTS)

None of the included studies used the surface EMG measurements to decide upon readiness for RTS (Table 4). However, the results from about a third of the studies (31.6%, 12 studies) could provide useful information by the choice of the assessed groups such as copers versus non-copers [33, 34, 47–49, 57], intervention and control group from the same team or level/league [35, 38, 40], data from pre-injury/pre-surgery including post-surgical follow up [62, 69] or participants with full RTS versus limited RTS [53]. In addition, two studies even investigated the influence of fatigue on neuromuscular control [41, 52].

More detailed information regarding EMG methods and procedures such as EMG type, detection, normalization, data processing and electrode placement can be found in Additional file “EMG methods and procedures of included studies” 3.

## Discussion

The aim of this systematic review was to summarize the scientific literature regarding EMG-related assessments for neuromuscular control in patients with an ACL injury (either treated surgically or conservatively). The second aim was to analyze whether these assessments for neuromuscular control were used to decide upon readiness for RTS in these patients.

There were many factors present which could have an influence on neuromuscular control.

### Influence by type of comparison (intra- versus inter-subject)

The use of the contralateral, non-injured leg in intra-subject comparison, without a “real” control group [42, 44] may lead to an overestimation of the physical performance in the ACL reconstructed or -injured leg. After ACLR, functional performance is often expressed with the LSI [70]. As the non-affected limb may also have deteriorated, the LSI may overestimate the right time for a safe RTS, and therefore, the risk for secondary injury may be higher [23]. In acutely injured ACL patients, intra-individual comparison showed bilateral consequences during stair ascent and indicates an alteration in the motor program (‘‘pre-programmed activity’’) [71]. In addition, in case of a case-controlled study design, the subjects in the control group should be matched to the ACL participants regarding sex, age, body mass, height, activity level and leg dominance.

### Influence by level of activity and fatigue

Some of the included studies used very challenging, sports-specific task to assess neuromuscular control, some even assessed neuromuscular control after fatiguing tasks. It is known that most of ACL tears are non-contact injuries happening at the end of a training session or a play [72]. Therefore, the closer the task to the sports and injury-risky situation, the safer the decision towards full RTS or even return to competition will be. However, assessing performance-based tests or movement quality may be more difficult to standardize, require more complex equipment and large amounts of space. But if only impairments will be tested, there will be a lack of information regarding an “athlete’s capacity to cope with the physical and mental demands of playing sport” [73]. It is therefore recommended to search for a standardized assessment close to the injury mechanism.

### Influence by gender

Not all included studies reported findings of mixed groups separately by gender. Some did not even state whether study participants were male or female. This could partly be explained by the date of publication as gender difference in ACL patients has not been in the focus of former ACL research. It is known that female athletes are more likely to sustain an ACL injury than men [74, 75]; the increased risk is probably multifactorial [76]. Several studies indicate that hormonal factors play a role [3, 77] contributing to an increased laxity of ligaments in the first half of the menstrual cycle. However, biomechanical and neuromuscular aspects as indicators are discussed controversially in literature: Gender-specific neuromuscular adaptations and biomechanical landing techniques are considered being the most important ones to explain the increased risk of injury in women [78, 79]. The higher risk for females to suffer from an ACL injury can be explained by motion and loading of the knee joint during performance [74]. Female athletes typically perform movements in sports with a greater knee valgus angle than men. Therefore, the amount of stress on the ACL in these situations is higher caused by a high activation of the quadriceps despite limited knee and hip flexion, greater hip adduction and a large knee adduction moment [80, 81]. The dominance of the quadriceps muscle in women could contribute to increased anterior tibial translation [82, 83] and was found in various activities such as jumps and cutting maneuvers [84–86]. Moreover, females typically land with an internally or externally rotated tibia [87], leading to an increased knee valgus stress due to greater and more laterally orientated ground reaction forces [83]. In contrast, other researchers did not find any gender-specific differences in the quadriceps-hamstrings ratio [88], not even in landing and cutting maneuvers [89]. A systematic review summarized biomechanical gender differences and stated that these were based on questionable clinical relevance [89]. In addition, strength-paired women and men showed no significant differences in neuromuscular activity [90].

### Influence by treatment

The included studies reported different treatment options (ACLR with different graft types, conservative treatment). Depending on the classification of the participants in copers and non-copers, the results in neuromuscular control may differ from a population of ACLR participants. Therefore, all researchers who worked with copers and non-copers made intra- and inter-group comparisons without an ACLR group. A Cochrane review revealed low evidence for no difference in young, active adults after two and five years after the injury, assessed with patient-reported outcomes. However, many participants described as “non-copers” with unstable knee with conservative treatments remain symptomatic, and therefore, later opt for ACL surgery [91]. It has been described that persistent co-contraction and joint stiffening in these “non-copers” is likely to be due to an abnormal neuromuscular strategy failing to restore joint stability in these ACL deficient group [92]. Furthermore, the choice of graft would influence the neuromuscular control of measured muscles due to the morbidity of the harvesting site of the graft (e.g. hamstrings).

### EMG variables

If the researchers mentioned the procedures for collecting EMG data, they referred to standardized applications and guidelines such as SENIAM (Surface Electromyography for the Non-Invasive Assessment of Muscles) [93]. The provided EMG-related variables were in accordance to the ones mentioned in a systematic review searching for knee muscle activity in ACL deficient patients and healthy controls during gait [14]. Current literature suggests greater co-contraction indices, increased joint stiffness and earlier muscle activation onset times as measures of neuromuscular function reflecting the incomplete restoration of normal joint stability [19, 92]. Some of the included studies reported values of muscle onset activity in milliseconds and percentage of gait cycle as a systematic review did by summarizing and quantitatively analyzing muscle onset activity prior to landing in patients after ACL injury [20]. However, no cut-off values out of EMG-related variables were provided to determine an adequate level of neuromuscular control. Moreover, some of the researchers only provided integrated EMG values which would make it difficult to be compared to other studies using the respective units (milliseconds, millivolts) or widely used percentage values (%MVIC, %MVC).

### Return to sports (RTS)

Regarding the determination of RTS after ACLR, there is some evidence for the use of functional performance tests, which had also been widely used in the included studies. Multiple functional performance measures—a battery including strength and hop tests, quality of movement and psychological tests [25]—might be more useful for the determination of RTS than a single performance measure. However, it is still unclear, which measures should be used to bring athletes safely back to RTS with a low risk of a second ACL injury [25]. Currently used RTS criteria or assessments, such as time, strength tests, hop tests, patient-reports, clinical examination, thigh circumference, ligamentous stability, range of motion, effusion and performance-based criteria, may be suboptimal at reducing the risk of a second ACL injury [73, 94]. Recovery of neuromuscular function was mentioned to be important because of the existing connection between the variables time since surgery and the risk for re-injury of the knee joint; but adequate assessment procedures to assess neuromuscular function are still a matter of debate [7]. In contrast, authors of an included study stated that “studies like ours that focus on the objective measurement of the change of the muscle latency time over time may allow patients to return to full activity and to sports earlier than the standard time of 6–12 months” [42]. However, this statement only based on one outcome measure and contrasts with current criterion- and time-based recommendations for RTS. Therefore, this recommendation seems to be rather dangerous.

## Limitations

The sample size of all the studies was quite low, however, providing reasonable sample size calculations and depending on the variable investigated, the results were acceptable. Furthermore, the more restrictive the inclusion criteria for the participants, the more homogeneous the intervention and the control groups were, but the more challenging the recruitment process was, leading to smaller groups to be investigated.

The used assessment for the risk of bias, the Downs and Black checklist [31] in a modified form [29, 32] is designed for randomized and non-randomized controlled studies, however, the latter score lower in some items, get lower total scores and therefore a worse overall rating of the methodological quality. Despite this disadvantage, we decided to use the modified checklist as we could assess all studies with different designs included in this systematic review. However, the use of total scores and choice of cut-off values for low, medium and high risk of bias, respectively, were arbitrary and not based on literature.

## Conclusions

### Implications for clinical practice

This systematic review summarized assessments using EMG variables for neuromuscular control of the knee in patients suffering from an ACL injury (either treated surgically or conservatively). Despite 38 articles providing a wide range of EMG-related assessments, none was used to decide upon readiness towards a safe and successful RTS in patients after an ACL injury. So far, there is no diagnostic measure to assess neuromuscular control and therefore, clinicians should use a multimodal approach including assessments for active and passive knee stability under different sports-related conditions but be aware of not being able to evaluate neuromuscular control in depth without EMG-related assessments. Moreover, the widely used LSI may overestimate the physical performance of an ACL patient as the non-affected limb is likely to have deteriorated, too.

### Implications for further research

Additional studies are needed to define readiness towards RTS by assessing neuromuscular control in adult ACL patients with EMG. Further research should aim at finding reliable and valid, EMG-related variables to be used as diagnostic tool for neuromuscular control. Due to the heterogeneity in participants, interventions and outcomes used, future studies should aim at more homogenous patient groups, evaluate females and males separately, provide adequately matched healthy subjects (gender, height, weight, activity level etc.), control for confounding factors such as type of treatment, and use tasks close to the injury mechanism, as sport specific as possible, respectively. Moreover, it would be interesting to assess not only lower leg but pelvic and core muscles in addition. This would help to give insight in the complex field of ACL injuries and subsequent rehabilitation strategies, and therefore improve knowledge towards a safe RTS in these patients.

## Supplementary Information

**Additional file 1** Search string for MEDLINE/PubMed.

**Additional file 2** Methodological quality assessment.

**Additional file 3** EMG methods and procedures of included studies.

## Data Availability

The datasets used and analyzed in the current study are available from the corresponding author on reasonable request.

## Abbreviations

ACL: Anterior cruciate ligament
ACLR: Anterior cruciate ligament reconstruction
EMG: Electromyography
LSI: Limb Symmetry Index
PEDro: Physiotherapy Evidence Database
PICOS: Participants-Intervention-Control-Outcome-Study design
PRISMA: Preferred Reporting of Items for Systematic reviews and Meta-Analyses
PROSPERO: International prospective register of systematic reviews
RTS: Return to sports
SENIAM: Surface Electromyography for the Non-Invasive Assessment of Muscles
TAS: Tegner Activity Score
