# Supplementary material for: Which assessments are used to analyze neuromuscular control by electromyography after an anterior cruciate ligament injury to determine readiness to return to sports? A systematic review
Source: BMC Sports Sci Med Rehabil. 2021 Nov 8;13:142. doi: 10.1186/s13102-021-00370-5 (PMC8577028; doi:10.1186/s13102-021-00370-5)
Supplement: Supplementary file 3 — Additional file 3 EMG methods and procedures of included studies. [file 13102_2021_370_MOESM3_ESM.docx]

**Additional file C: EMG methods and procedures of included studies**

**Belonging to the manuscript entitled**

**Which assessments are used to analyze neuromuscular control by electromyography after an anterior cruciate ligament injury to determine readiness to return to sports? A systematic review**

Angela Blasimann^a,b,^*, MSc, Irene Koenig^a^, PhD, Isabel Baert^b^, PhD, Heiner Baur^a^, Prof., Dirk Vissers^b^, Prof.

^a^Bern University of Applied Sciences, Department of Health Professions, Division of Physiotherapy, Murtenstrasse 10, Bern CH-3008, Switzerland

^b^University of Antwerp, Faculty of Medicine and Health Sciences, Department of Rehabilitation Sciences and Physiotherapy, Campus Drie Eiken, Universiteitsplein 1, Wilrijk B-2610, Belgium

*corresponding author

EMG methods and procedures of included studies

| **Authors & Year** | **Type** | **Detection** | **Electrode placement** | **Sampling** | **Rectification** | **Post-Processing** | **Normalization** |
| --- | --- | --- | --- | --- | --- | --- | --- |
| Busch et al. (2019) (13) | sEMG | bipolar | according to SENIAM (Hermens et al., 2000 (93)) | differential-preamplifier (gain: 500, input impedance: 4000 M, common mode rejection ratio 90 dB at 60 Hz) to a telemetric main amplifier (band-pass filter: 10 Hz to 1 kHz, gain: 5.0, resultant overall gain: 2500)  sampling rate: 2000Hz | n.m. | band-pass filter 10–500 Hz (Butterworth, 2^nd^ order) | to the individual, submaximal MVC during walking on treadmill at 5 km/h (1.39 m/s) (%subMVC) |
| Alkjaer et al. (2003) (33) | sEMG | bipolar | 2 cm apart over the most prominent  part of the muscle belly | custom-built preamplifiers (input impedance 80 MΩ, gain=50), custom-built amplifiers with a frequency response between 20 Hz and 10 kHz  sampling rate: n.m. | full-wave | digitally high- and low-pass filtered (Butterworth 4^th^ order, cut-off frequencies  20 Hz and 500 Hz, respectively), low-pass filtered at 15 Hz | to the maximal EMG amplitude (%maxEMG) recorded during  MVIC for each muscle  group |
| Alkjaer et al. (2002) (34) | sEMG | bipolar | n.m. | custom-built preamplifiers (input impedance 80 MΩ, gain=50) with a frequency response between 20 Hz and 10 kHz  sampling rate: 1000 Hz | full-wave | digitally high- and low-pass filtered  (Butterworth 4^th^ order, cut-off frequencies  20 Hz and 500 Hz, respectively), low-pass filtered at 15 Hz | n.m. |
| Arnason et al. (2014) (35) | sEMG | bipolar | according to SENIAM (Hermens et al., 2000 (93)) & palpation during muscle contraction | signal bandwidth of 16–500 Hz  sampling rate: 1600 Hz | full-wave | high-pass filtered at 25 Hz  moving 250 ms window | to the maximum signal collected during  3 MVIC,  each lasting 5 s |
| Bryant et al. (2009) (36) | sEMG | bipolar | over the relevant  muscle bellies according to Daanen et al., 1990 & De Luca,  1997 | amplifier (gain 10000; common mode rejection  >120 dB; input impedance >1012 Ω), following amplification (gain = 10000; common mode rejection >120 dB; input bias current <40 pA; input impedance >1012 Ω)  sampling rate: 1000 Hz | full-wave | 4^th^ order zero-phase-shift Butterworth  filter (Winter, 1990; high-pass fc = 15 Hz; low-pass  fc = 250 Hz) for linear envelope, after rectification: 4^th^ order zero-phase-shift Butterworth low-pass  filter (fc = 30 Hz). | n.m. |
| Burland et al. (2020) (37) | sEMG | bipolar | n.m. | n.m. | yes, not further specified | high-pass Butterworth filter using a  12.0 Hz cut-off frequency, after rectification processed using a RMS algorithm with a 50 ms  moving window | EMG data from single-limb forward hop normalized to peak muscle activity recorded across all trials |
| Cordeiro et al. (2015) (38) | sEMG | bipolar | according to SENIAM (Hermens et al., 2000 (93)) | amplified with a band-pass (10–500 Hz), common mode rejection ratio (120 dB) and input impedance  > 100 MW  sampling rate: 1050 Hz | full-wave | digitally filtered (20–500 Hz), after rectification low-pass filter  (25 Hz, 7^th^ order Butterworth) | to amplitude expressed as RMS obtained during MVIC |
| Dashti Rostami et al. (2019) (39) | sEMG | bipolar | according to SENIAM (Hermens et al., 2000 (93)) | sampling rate: 2000 Hz | full-wave | band-pass filtered using 4^th^ order, zero-lag Butterworth filter with high- and low-pass  cut-off frequencies of 10 and 500 Hz, respectively, after rectification: using RMS-algorithm with 50ms moving window. | dynamic EMG data from landing task normalized to the  peak muscle activity recorded during MVIC |
| Jordan et al. (2016) (40) | sEMG | bipolar | according to SENIAM (Hermens et al., 2000 (93)) | Preamplification (overall gain 500), filtered with 1^st^ order high-pass filter (10 Hz) and low-pass filtered (cut-off 500 Hz), common mode rejection ratio  >100 dB  sampling rate: 1500 Hz | n.m. | high-pass filtered (cut-off frequency = 10 Hz) using Butterworth 4^th^ order zero-lag filter, smoothed using a point-by-point moving 50 ms symmetric RMS filter | RMS for each muscle during jump test normalized to the maximal RMS amplitude obtained from respective  muscle during MVIC |
| Lessi et al. (2017) (41) | sEMG | bipolar | according to SENIAM (Hermens et al., 2000 (93)) | preamplification (operating range 40 m, transmission frequency  2.4 GHz, common mode rejection ratio >80 dB; bandwidth of 450 Hz at >80 dB/s)  sampling rate: 2400 Hz | full-wave | band-pass  filtered at 20–400 Hz, smoothed by symmetrical moving RMS filter (20 ms time constant) | to peak RMS amplitude recorded during landing, representing 100% activity (%peak EMG during landing) |
| Oliver et al. (2018) (42) | sEMG | bipolar | according to SENIAM (Hermens et al., 2000 (93)) | amplification and band-pass-filter in working frequency of 1000 Hz | n.m. | n.m. | by setting RMS amplitude at a percentage related to the MVC (%MVC) |
| Ortiz et al. (2014) (43) | sEMG | bipolar | according to Cram et al., 1998 | signal bandwidth: 20-450 Hz [3 dB]; impedance:  >100 KU; noise: <1.2 mV  amplified (gain = 1000)  sampling rate: 1000 Hz | n.m. | band-pass filter (20-450 Hz) | by dividing the mean signal during each specific task  trial by the maximum signal generated during the middle  3 seconds of the 5-second tuck jumps performed during  warm-up |
| Patras et al. (2009) (44) | sEMG | bipolar | according to SENIAM (Hermens et al., 2000 (93)) and Merletti & Hermens, 2004 | signal bandwidth: 10–500 Hz  sampling rate: 1500 Hz | full-wave | high-pass filtered with an 8^th^ order Butterworth filter, smoothed with a 100 ms RMS algorithm | n.m. |
| Patras et al. (2010) (45) | sEMG | bipolar | according to SENIAM (Hermens et al., 2000 (93)) and Merletti & Hermens, 2004 | signal bandwidth: 10–500 Hz  sampling rate: 1500 Hz | full-wave | high-pass filtered (cut-off  frequency at 20 Hz) with an 8^th^ order Butterworth filter, smoothed with a 100 ms RMS algorithm | not performed |
| Pincheira et al. (2018) (46) | sEMG | bipolar | according to SENIAM (Hermens et al., 2000 (93)) | signal bandwidth of 20-450 Hz  sampling rate: 1000 Hz | full-wave | digital low-pass filtered  at 50 Hz | n.m. |
| Rudolph et al. (2001) (47) | sEMG | bipolar | n.m. | sampling rate: 960 Hz | full-wave | low-pass filter (2^nd^ order, phase corrected, Butterworth  filter) with cut-off frequency of 20 Hz | to a maximum  EMG, which was defined as the highest level of EMG over a  30-ms interval during any of the isometric, walking or jogging trials (%MVIC) |
| Rudolph et al. (2000) (48 | sEMG | bipolar | n.m. | n.m. | full-wave | low pass filter (2^nd^ order, phase corrected, Butterworth  filter) with cut-off frequency of 20 Hz | peak EMG activity over 30-ms from either the dynamic or maximum  isometric trials used to normalize EMG data in dynamic trials |
| Rudolph & Snyder-Mackler (2004) (49) | sEMG | bipolar | over the muscle bellies according to Delagi et al., 1981 | sampling rate: 960 Hz | full-wave | phase corrected, 8^th^ order, Butterworth  filter, with high-pass cut-off frequency of 90 Hz, after rectification: low-pass filter with 2^nd^ order, phase corrected, Butterworth filter with cut-off frequency of 20 Hz | maximum EMG defined as highest amplitude of EMG found during any of the trials (MVIC and dynamic tasks). Signals from step trials were then normalized by maximum value |
| Swanik et al. (2004) (50) | sEMG | bipolar | identified by bony landmarks  and by palpating midlength of the contractile component  during isometric contraction | single-ended amplifier (gain 500) with 4^th^ order Butterworth filter (10–500 Hz), common mode rejection ratio of 130 db.  sampling rate: 2500  Hz | full-wave | smoothing over a 15-ms moving window | peak muscle activity during landing used for amplitude  normalization |
| Briem et al. (2016) (51) | sEMG | bipolar | according to SENIAM (Hermens et al., 2000 (93)) | signal bandwidth  of 16–500 Hz  sampling rate: 1600 Hz | full-wave | high-pass filtered at  25 Hz, RMS derived using moving 250 ms window | peak values test jump normalized to maximum signal of two 5-s trials of MVIC (%MVIC) |
| Lessi et al. (2018) (52) | sEMG | bipolar | according to SENIAM (Hermens et al., 2000 (93)) | preamplification (transmission frequency  2.4 GHz, common mode rejection ratio >80 dB; bandwidth of 450 Hz at >80 dB/s)  sampling rate: 2400 Hz | full-wave | band-pass  filtered at 20–400 Hz, smoothed by symmetrical moving RMS filter (20 ms time constant) | against peak RMS during landing phase 🡪 peak RMS during landing represented 100% of muscle activity (%peak RMS during landing task) |
| Lustosa et al. (2011) (53) | sEMG | bipolar | on the largest portion of the VL and BF  muscles, according to the direction of the fibers | amplifiers with input impedance of 2 MΩ, common mode rejection ratio of 1000 MΩ  signal bandwidth: 10 to 2000 Hz  built-in high-gain amplifier with an integral 500 Hz low-pass filter  sampling rate: 1000 Hz | full-wave | filtered with 500-Hz low-pass and 10 Hz high-pass  filters | RMS used to quantify intensity of EMG signal during  MVIC tests 🡪 values used to normalize  the EMG signal (%MVIC) |
| Nyland et al. (2010) (54)  Nyland et al. (2013) (55)  Nyland et al. (2014) (56) | sEMG | bipolar | applied in parallel alignment to the  muscle fibers at muscle belly | signal bandwith: 10 - 500 Hz, differential input impedance >10 MΩ, common mode rejection ratio of 100 dB at 50 or 60 Hz  sampling rate: 1000 Hz | full-wave | 60-Hz notch filter and 50 ms RMS-smoothing | mean EMG signal amplitudes collected  during single-leg CMJ testing (54,55), single-leg hop for distance (56) respectively, normalized to MVIC |
| Boerboom et al. (2001) (57) | sEMG | n.m. | according to Perotto, 1994 | n.m. | yes, not further specified | band-pass filtered 20 Hz–10 kHz and smoothed with a 25 Hz 3^rd^ order Butterworth filter. Smoothed rectified EMGs were A/D converted at 100 Hz | individually averaged  EMG pattern normalized to standard pattern of healthy control group (normative EMG profiles), several steps of calculations |
| Bulgheroni et al. (1997) (58) | sEMG | n.m. | n.m. | sampling rate: 500 Hz | yes, not further specified | High-pass filter to eliminate frequency components <10 Hz, after rectification: filtered to  eliminate the components of the signal > 200 Hz | to the maximum recorded signal amplitude during a single walking cycle |
| Gokeler et al. (2010) (59) | sEMG | bipolar | according to SENIAM (Hermens et al., 2000 (93)) | preamplifier with >110 dB common mode rejection, <2 microV RMS noise level, >500MΩ input impedance  high-pass filter at 20 Hz with 3^rd^ order digital  Butterworth filter  sampling rate: 800 Hz | yes, not further specified | Smoothing with 10 Hz  zero-lag Butterworth filter | n.m. |
| Hansen et al. (2017) (60) | sEMG | n.m. | according to SENIAM (Hermens et al., 2000 (93)) | sampling rate: 2000 Hz | n.m. | high-pass filter with 5 Hz cut-off, low-pass with 500 Hz cut-off | normalized to its respective maximal contraction value during 100% body weight running trials for each participant |
| Klyne et al. (2012) (61) | sEMG | n.m. | according to Kendall and McCleary, 1983 | signal bandwidth of 20–450 Hz ±10% (incorporated in electrodes)  preamplifier (gain = 1000 V/V)  differential input voltage range of ±10 V, resolution of 100 mV, max sampling rate of 100 kS/s, max gain of x500, common mode rejection rate 105 dB, input impedance of 1 GΩ,  sampling rate: 1000 Hz | n.m. | 2^nd^ order, low-pass, Butterworth filter with cut-off frequency of 500 Hz | RMS collected during single leg hop test normalized to peak MVIC RMS recordings over a 150 ms epoch |
| Knoll et al. (2004) (62) | sEMG | bipolar | on the skin overlying the muscle belly | sampling rate: 1000 Hz | yes, not further specified | high-pass filter to eliminate  frequency components <10 Hz, after rectification: filtered to  eliminate the components of the signals > 200 Hz  RMS and normalized | to average of peak EMG signal values of six gait cycles 🡪 EMG patterns during % of gait cycle |
| Kuster et al. (1995) (63) | sEMG | bipolar | n.m. | amplified, band-pass  filtered (3 dB down at 3 Hz and 1 kHz)  sampling rate: 500 Hz | full-wave | high-pass filtered  at 4 Hz to eliminate movement artefacts | averages of 10-19 strides for each subject 🡪 representative group linear envelope of these averaged EMG records obtained by across-subject averaging following normalization of each individual’s  ensemble average to 100% of peak activity |
| Madhavan & Shields (2011) (64) | sEMG | bipolar | according to Cram et al., 1998 | electrodes with on-site preamplification  (gain x 35), further amplified at main frame by 10 K (high-impedance circuit with common mode rejection ratio of 87 dB at 60 Hz)  signal bandwidth: 15–4000 Hz  sampling rate: 2000 Hz | n.m. | calculation of linear velocity by differentiating displacement signal (Tc = 10 ms) and low-pass filtering at 6 Hz using a 5^th^ order zero phase lag Butterworth filter  RMS processed with a time constant of 10 ms | analysis of MVICs by  finding peak RMS during each muscle contractions 🡪 calculating mean RMS for 200ms on either side of the peak EMG (%MVIC) |
| Ortiz et al. (2008) (65)  Ortiz et al. (2011) (66) | sEMG | bipolar | according to Cram et al. 1998 | preamplified electrodes, overall gain 2000 mV  filtered at a bandwidth of 10 to 500 Hz with 130 dB common-mode rejection within the transmitter  sampling rate: n.m. | full-wave | n.m. | by using a dynamic normalization procedure in which the mean signal for  each muscle group in the window of interest was divided by the maximum signal generated on the specific trial analyzed |
| Patras et al. (2012) (67) | sEMG | bipolar | according to SENIAM (Hermens et al., 2000 (93)) and Merletti & Hermens, 2004 | signal bandwidth: 10–500 Hz  sampling rate: 1500 Hz | full-wave | high-pass filtered (cut-off  frequency at 20 Hz) with an 8^th^ order Butterworth filter, smoothed with a 100 ms RMS algorithm | not performed |
| Swanik et al. (1999) (68) | sEMG | bipolar | according to Basmajian & De Luca, 1985 | amplifier (gain 500) with Butterworth low-pass (15  Hz) and high-pass (500 Hz) filters, common mode rejection ratio of 130 dB, receiver (gain 500, total gain 1000)  sampling rate: 2500 Hz | full-wave | integrated EMG data averaged over a 15 ms moving window with sampling rate of 1000 Hz | integrated EMG (microvolts x ms) normalized to mean amplitude of 3 - 6 consecutive test repetitions and for time |
| Zebis et al. (2017) (69) | sEMG | bipolar | according to SENIAM (Hermens et al., 2000 (93)) | n.m. | n.m. | high-pass filtered at a 5Hz cut-off frequency (4^th^ order zero-lag Butterworth filter), subsequently smoothed by symmetrical moving RMS filter of 30 ms | mean RMS amplitude normalized to peak RMS amplitude recorded during sidecutting maneuver (%maxEMG) |

Legends: CMJ = countermovement jump(ing); EMG = electromyography; MVC = maximum voluntary contraction; MVIC = maximum voluntary isometric contraction; Hz = Hertz; ms = milliseconds; n.m. = not mentioned; RMS = root mean square; sEMG = surface electromyography; SENIAM = Surface ElectroMyoGraphy for the Non-Invasive Assessment of Muscles Project

Additional references (in order of apperance):

Daanen HA, Mazure M, Holewijn M, Van der Velde EA. Reproducibility of the mean power frequency of the surface electromyogram. Europ J Appl Physiol Occup Physiol. 1990;61(3–4):274–7.

De Luca CJ. The use of surface electromyography in biomechanics. J Appl Biomech. 1997;13:135–63.

Winter DA. Biomechanics and motor control of human movement. 2nd ed. New York: Wiley; 1990.

Cram JR, Kasman GS, Holtz J. Introduction to Surface Electromyography. Aspen: Gaithersburg; 1998.

Merletti R, Hermens HJ. Detection and conditioning of the surface EMG signal. In: Merletti R, Parker P (eds). Electromyography: physiology, engineering and non-invasive applications. New Jersey: Wiley; 2004.

Basmajian JV, DeLuca C. Muscles Alive: Their Functions Revealed in Electromyography. 5th ed. Baltimore: Williams & Wilkins; 1985.

Delagi EF, Iazzetti J, Perotto A, Morrison D. Anatomical Guide for the Electromyographer. Springfield: Charles C. Thomas; 1981.

Perotto AO. Anatomical guide for the electromyographer. The limbs and the trunk, 3rd ed. Springfield: Thomas; 1994.

Kendall FP, McCleary EK. Muscles: testing and function. Baltimore, USA: Williams and Wilkins; 1983
